# Effect of Trataka on Visual, Cognitive, and Psychophysiological Outcomes: A Systematic Review and Meta-Analysis

**DOI:** 10.3390/healthcare14213624

**Published:** 2026-10-26

**Authors:** Sowmiya Krishna, Sandeep Bodda, Sushma Naranappa Salethoor, Shyamasundaran Kulangara, Rammanohar Puthiyedath, Veena Nair, Shyam Diwakar

**Affiliations:** 1 Amrita Mind Brain Center, Amrita Vishwa Vidyapeetham, Amritapuri, Kollam 690525, Kerala, India; am.ls.r4ccn23004@am.students.amrita.edu (S.K.); sandeepb@am.amrita.edu (S.B.); 2 Department of Shalakya Tantra, Amrita School of Ayurveda, Amrita Vishwa Vidyapeetham, Amritapuri, Kollam 690525, Kerala, India; sushma@ay.amrita.edu; 3 Department of Samhita, Sanskrit and Siddhanta, Amrita School of Ayurveda, Amrita Vishwa Vidyapeetham, Amritapuri, Kollam 690525, Kerala, India; shyamasundarank@ay.amrita.edu; 4 Amrita Centre for Advanced Research in Ayurveda, Amrita School of Ayurveda, Amrita Vishwa Vidyapeetham, Amritapuri, Kollam 690525, Kerala, India; rammanohar@ay.amrita.edu; 5 Department of Radiology, University of Wisconsin-Madison, Madison, WI 53705, USA; vnair@uwhealth.org; 6 Department of Electronics and Communication Engineering, Amrita Vishwa Vidyapeetham, Amritapuri, Kollam 690525, Kerala, India

**Keywords:** Trataka, yoga, cognition, visual attention, neuroscience, SDG 3, complementary therapy

## Abstract

**Highlights:**

- Trataka studies show a preliminary, statistically insignificant trend suggesting longer practice durations may be associated with higher cognitive scores, requiring confirmation through larger studies.
- As a healthcare technique, Trataka shows preliminary evidence of cross-modal neurocognitive modulation across visual, cognitive, and psychophysiological domains.
- High heterogeneity-centric studies underscore the need for an integrated, evidence-based public health approach to Trataka that ensures reliability, replicability, and reproducibility across ages, and evaluates duration–response correlations.

**Abstract:**

Objective: A complementary and alternative healthcare practice such as Trataka, a Yogic visual concentration technique (YVC) rooted in Ayurvedic tradition, links cognition, visual, and psychophysiological well-being. This meta-analysis aimed to evaluate the moderating factors, including variations in study design and intervention protocol, to understand the efficacy of YVC across multiple domains. Methods: Systematic searches of eight scientific databases obtained 34 studies from 280 records until December 2025. Based on task scores, meta-regression and statistical heterogeneity were assessed using a random-effects model to obtain standardized mean difference (SMD) and risk of bias (RoB). Results: A pooled meta-analysis showed a significant YVC effect over control conditions across visual, cognitive, and psychophysiological domains (SMD of 0.92, 95% CI [0.44, 1.41]), with substantial heterogeneity (I^2^ = 74%) and a wide prediction interval (−0.22 to 2.07), which indicated that the true effect size could range from negligible to large, while extending to future studies. Meta-regression across the nine cognitive studies (K = 4 for each study design; k = 1 outlier) revealed no statistically significant duration–response relationship between session length and cognitive outcomes. RoB assessments indicated unclear to high risk among included studies. Discussion: Our results indicate that regular YVC practice could be associated with potential benefits for ocular and cognitive wellbeing, and for stress reduction, aligned with Sustainable Development Goal 3. However, given substantial heterogeneity, methodological limitations, and RoB concerns among included studies, these findings should be interpreted cautiously. Standardized protocols, larger controlled trials, and neuroimaging approaches are needed to more precisely characterize YVC-related effects.

## 1. Introduction

Trataka, a structured yoga-derived attentional practice involving sustained time-limited focus aimed at strengthening visual attention over time [1,2,3], is referred to as the Yogic visual concentration technique (YVC) in this study [4,5]. A type of complementary therapy rooted in Ayurveda [6] that uses visual concentration, based on yogic principles and neurophysiological studies [7]. Many complementary therapy practices in yoga, including Asana (exercises) [8], Pranayama (breathing exercises) [9], and Dhyana (meditation) [10], have been recognized for their role in changing people’s lifestyles and improving their mental health [11,12,13].

More than 2.2 billion people globally are reported to suffer from various forms of vision impairment, according to the World Health Organization [14]. Following the COVID-19 pandemic [15], the increased use of digital screen time has led to eye strain [16] and vision problems such as myopia, diabetic retinopathy, glaucoma, and age-related macular degeneration [17,18]. Increased online learning and remote working have raised global health concerns [19], underscoring the importance of preventive methods to reduce the impact of ocular diseases that aid in achieving Sustainable Development Goal 3 (SDG3) [20]. With the growth of digitally reliant sedentary lifestyles, complementary therapies are being evaluated to meet the new challenges [9,21]. Studies [22,23,24] have shown that traditional methods in mental resilience address the growing burden through non-pharmacological, cost-effective, and sustainable approaches [16,25]. While measuring traditional techniques, such as YVC limited information is available in terms of its neurophysiological correlates [26,27]. However, there has been limited focus on visual attention and cognitive effects related to YVC. To tackle these challenges, there is a need to understand the neuroscientific basis of visual attention in terms of cognitive health [28]. Neuroscience research has been interested in exploring the neural mechanisms underlying attentional control [29,30] and perceptual processing [31] to develop sustainable treatment strategies [32].

Visual attention is one of the essential cognitive-functional domains [33,34,35,36,37,38,39] that enables individuals to focus on significant details while selectively ignoring distractions [40,41] through both top-down (cognitive) and bottom-up (stimulus-driven) processes [42,43,44]. Different forms of visual attention have been reported in studies, involving selective attention [45], divided attention [46], and sustained attention [47], which includes YVC, providing valuable insights into how attentional control influences perception and higher-order cognitive processes [48,49,50,51] that are mediated by distributed cortical and subcortical networks [52]. Studies [29,53,54] have proposed that YVC practice effects have a role in the neural basis for sustained attention and cognitive modulation, activating higher-order visual regions from V1 to V5 [7,55,56] involving ipRGCs, which transmit signals beyond the primary visual system [57,58].

In meditation research, studies have hypothesized modulation of the default mode network (DMN) [59], where reduced DMN activity during focused attention [60] was generally associated with decreased mind-wandering [61] and improved task engagement [34,60]. Studies have indicated that sustained visual fixation [62,63] was linked to oculomotor and attentional regulation [64], recruiting neural circuits related to the frontal eye fields [65] and superior colliculus [37,66]. Theta and alpha oscillatory activity in the prefrontal and occipital regions [41,49] during sustained-attention tasks is associated with default-mode [61] and salience-network connectivity [53]. Related work [67,68,69] has linked sustained visual cues to attention regulation and cortical plasticity, with cortical mapping studies having reported changes in visual, executive, and emotion-regulation regions in the cortex following sustained practice [56,70]. In general, yoga and meditative studies [71,72,73] have used neuroimaging methods including fMRI [74,75,76], EEG [77,78,79], and MEG [80] for brain-mapping [81], while microstate-based network analyses provide an additional framework for understanding multi-scale brain organization [82,83,84]. Despite the lack of data supporting pathways for visual and cognitive processes, YVC has been characterized as a low-cost, non-invasive, and accessible practice [85,86] with potential to improve eye health, attention, and stress reduction, aligning with public health objectives [87,88].

Evidence from the YVC studies on direct neurophysiological assessment of YVC practice remains limited. Short-term practice has been associated with reduced EEG entropy across frontal, temporal, and occipital regions, indicative of altered cortical activity patterns [66], and with an increase in Critical Flicker Fusion frequency, suggesting enhanced cortical arousal [5]. Improvements in shooting accuracy following structured YVC technique have similarly been interpreted as evidence of enhanced oculomotor coordination and visual-motor integration [89]. However, a previous study on visual evoked potential (P100) latency had reported no significant change among short-term practitioners [90]. YVC’s effects across health-related domains remain to be addressed in the context of brain function and processing of oculo-sensorimotor functions. Classical texts describing YVC, including the Gheraṇḍa Saṃhitā and Haṭha Yoga Pradīpikā [1], do not specify a standardized, step-by-step protocol for duration, frequency, gaze-target characteristics, or progression, and contemporary studies consequently vary in these parameters as well as in visual cues and outcome measures [91,92,93], contributing to substantial heterogeneity that limits reproducibility. No prior meta-analysis has quantitatively synthesized YVC’s effects across visual, cognitive, and psychophysiological domains while accounting for this methodological diversity.

The use of RoB in evaluating the literature suggested a lack of unified assessment of how YVC may influence distinct cognitive domains such as attention, working memory, processing speed, and inhibitory control. Owing to its simplicity, minimal resource requirements, and ease of practice, YVC is being explored as a feasible attentional training approach across diverse populations and settings. This study assesses the methodological efficacy of YVC techniques across diverse study designs to explore how YVC can contribute to sustainable health practices. Integrative approaches to lifestyle management such as YVC that align with SDG3 can ensure healthy lives and promote well-being for all ages [94].

This preliminary meta-analysis aims to achieve the following:

1. Evaluate the pooled effects of YVC on cognitive, visual, and psychophysiological outcomes compared with control or baseline conditions, and characterize the methodological quality, study-level heterogeneity, and risk of bias underlying this evidence across between-subject and within-subject study designs.

2. Examine, on a preliminary basis, whether session duration can act as a potential moderator of cognitive effect size through meta-regression analysis.

## 2. Materials and Methods

This meta-analysis followed the Preferred Reporting Items for Systematic Reviews and Meta-Analyses (PRISMA) 2020 guidelines (see Supplementary Table S8) [95]. Eligibility criteria and the analytic approach were defined by the authors before study selection and data extraction. Prospective registration for the review protocol was not undertaken with PROSPERO or any other registry. As the study did not involve animal or human participants, no ethical approval was required. Only studies published in English or accompanied by a comprehensive English abstract were considered. Titles, abstracts, and full texts were independently screened by all authors. Given the limited number of available studies on YVC, no formal reviewer agreement was required. All discrepancies were resolved through discussion until a general agreement was achieved.

### 2.1. Search Strategy

A systematic search was performed across eight electronic databases, including PubMed, Scopus, Web of Science, Google Scholar, Cochrane Library, DOAJ, ProQuest, and JSTOR, to identify articles related to Trataka or YVC. The search strategy combined practice-related terms (Trataka; Yogic visual concentration; Yogic gazing) with domain/outcome terms (cognition; visual attention; EEG; fMRI; physiology; neural mechanisms; visual pathways). These term sets were used with Boolean operators as detailed in Table 1. No records were identified prior to November 2010. No field restrictions or limits were applied when retrieving records, and no predefined stopping criterion was used for the Google Scholar search. From 280 studies, 34 met the inclusion criteria and were included in the analysis. Eligible studies included publications in English up to December 2025 and included research articles, conference papers, dissertations, and relevant academic web sources.

**Figure 1:**
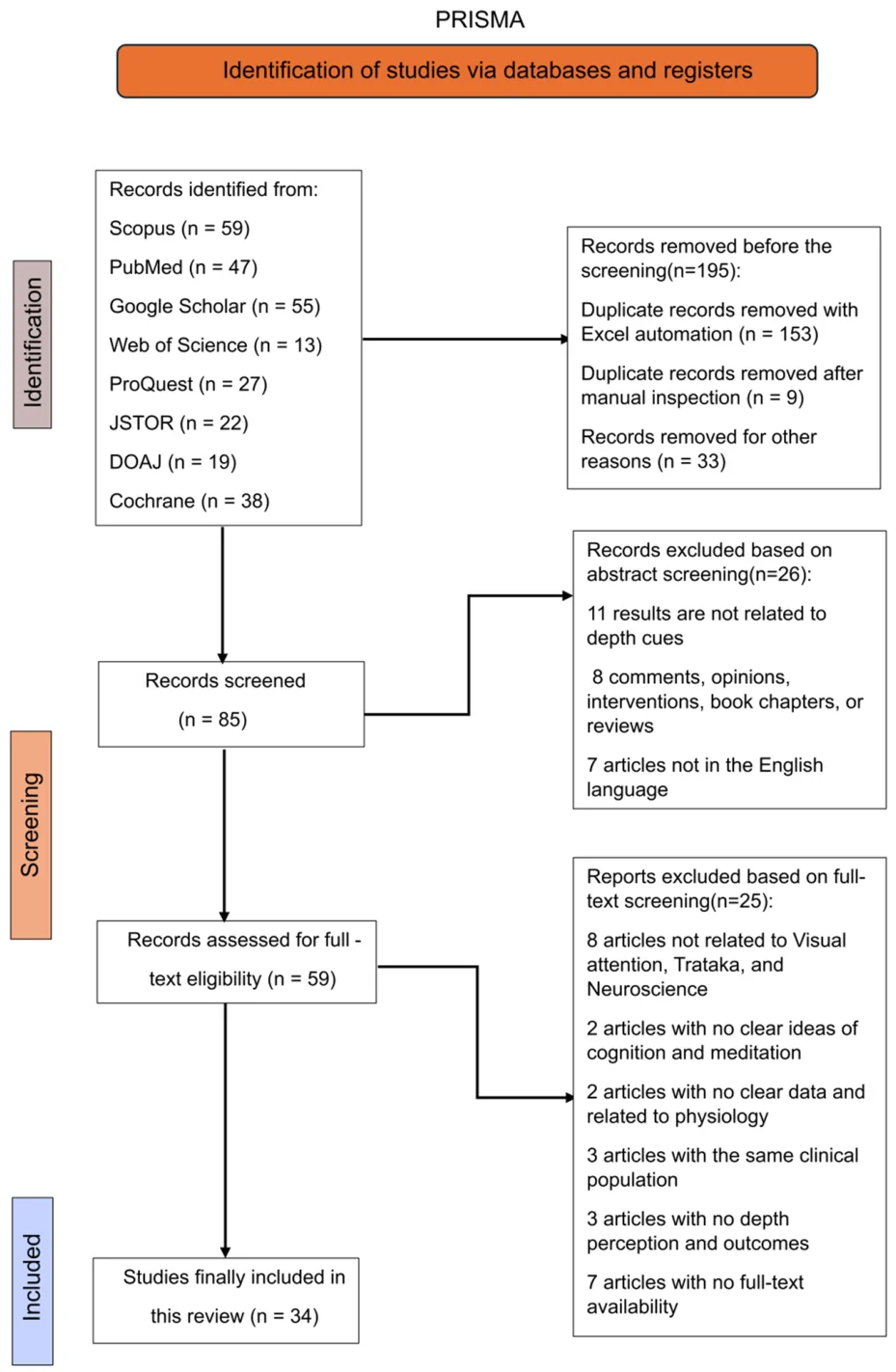
The schematic of the selection process included the identification, screening, eligibility, and inclusion of studies.

The PRISMA 2020 flow diagram illustrated the identification, screening, eligibility assessment, and inclusion of studies in the meta-review on YVC and its effects on cognition and visual attention-related outcomes. Out of 280 records, 195 duplicates were removed before screening, yielding 85 records. From 85, 34 studies were included in this review (see Figure 1).

### 2.2. Inclusion and Exclusion Criteria

This systematic review and meta-analysis were conducted using the PICOS framework (Population, Intervention, Comparators, Outcomes, and Study designs) [96] to ensure methodological clarity and transparency (Supplementary Table S1). The study design includes randomized controlled trials, quasi-experimental designs, pre–post intervention studies, and within-subject repeated measures that precisely evaluated the effects of YVC.

#### 2.2.1. Studies Included

Population: The review focused on human participants of all age groups, including children, adults, and older adults, regardless of their prior experience with yoga or meditation practices.

Intervention: YVC is practiced as a standalone technique. Studies in which YVC was combined with other yoga or meditation methods were included only if YVC-specific effects were reported as a separate outcome.

Comparison: YVC was composed of no-intervention or waitlist groups, routine daily activities, or standard care, using within-subject designs and participants’ own baseline measures.

Outcome: Cognitive functions such as attention, memory, processing speed, and executive function. Emotional or psychological parameters, including stress, anxiety, mood, or sleep quality. Visual health, including digital eye strain, refractive symptoms, visual comfort, or ocular fatigue. Physiological markers, such as heart rate, blood pressure, and insomnia symptoms. Neurophysiological responses, such as EEG, fMRI, or other indices related to brain activity. Published in English or with a comprehensive English abstract that provided sufficient methodological and results information for analysis.

#### 2.2.2. Studies Excluded

Lack of empirical data: review articles, conceptual papers, editorials, commentaries, or anecdotal accounts without primary data or statistical analysis.

Intervention ambiguity: Combined YVC with other interventions (e.g., Pranayama, general Yoga) without isolating YVC-specific effects or reporting them as a distinct intervention.

Unrelated outcomes: Outcomes that are not relevant to cognition, emotion, vision, or physiology, or are focused on unrelated physical health markers (e.g., cholesterol, diabetes, musculoskeletal pain).

Duplicate or redundant data: Repeated or overlapping data from the same research group already included in the review, unless new outcome domains or participant groups were analyzed.

Methodological limitations: Lack of baseline or comparison condition or sample size too small (*n* = 3) to permit statistical analyses.

### 2.3. Data Extraction and Management Procedures

**Figure 2:**
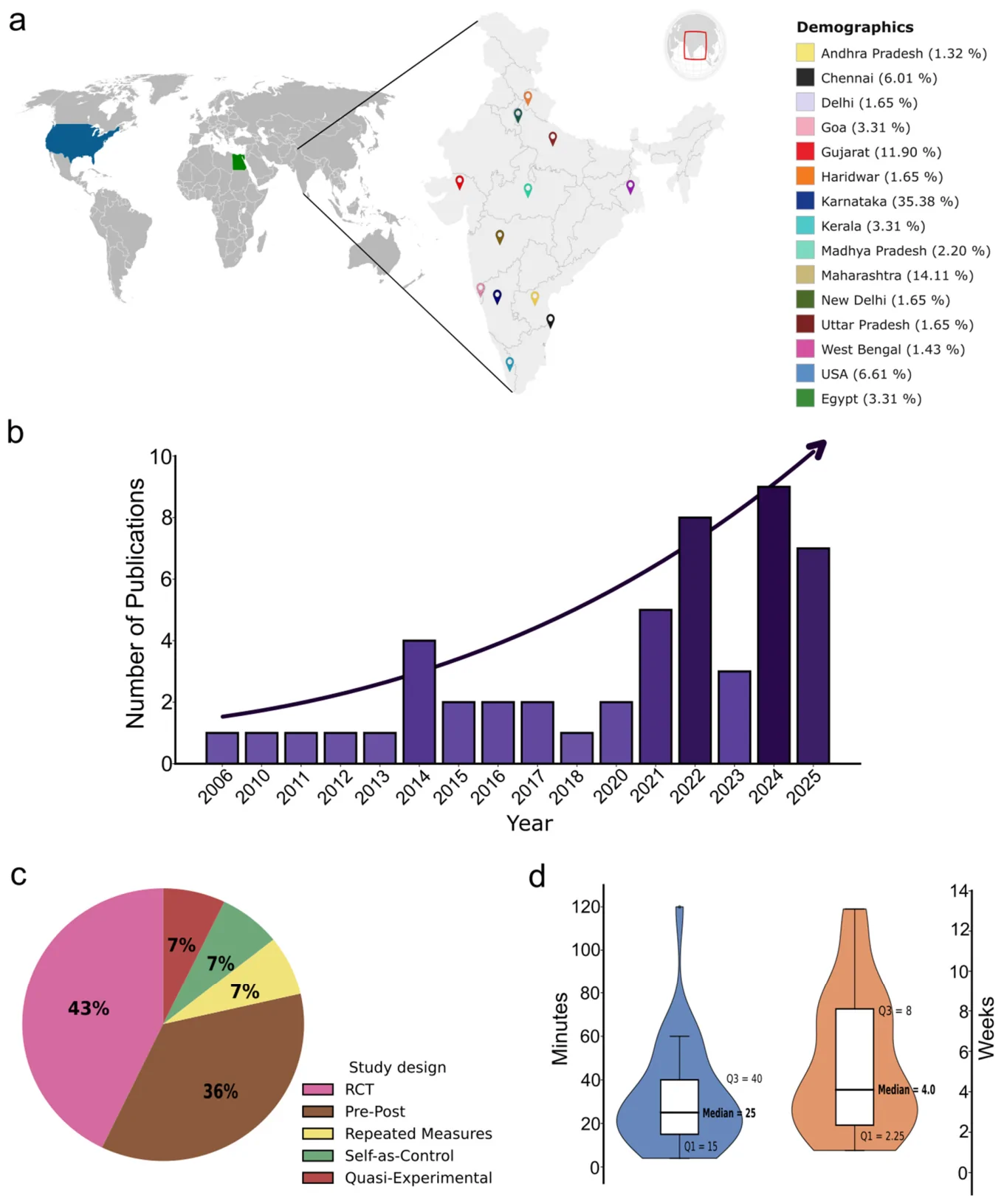
Characteristics of YVC research studies by intervention time and study design. (**a**) Geographic spread and diversity of the participant pool, along with the percentage. The map shows the countries of origin of participants included in the meta-analytic review. Most cohorts were drawn from different regions across India, with additional groups contributing from the United States and Egypt. (**b**) Research trend of YVC studies on the Y-axis, published between 2006 and 2025 on the X-axis. Early years showed sparse activity, with a sharp rise in 2014 and a rapid increase from 2010 onwards. The highest publication counts (purple) were observed in 2022 and 2025, showing a growing scientific interest in YVC research in recent years. (**c**) Distribution of study designs within the cognition domain. Randomized controlled trials (RCTs) were the most common (pink-43%), followed by pre–post studies (brown-36%), while quasi-controlled (red), self-as-control (green), and repeated-measures designs (yellow) each account for 7% of the included studies. (**d**) Box-and-violin plots of YVC practice parameters indicate the session duration and training duration values, with the embedded box plot showing the interquartile range of the data points (Q1–Q3), with the median indicated by a horizontal line across all studies.

Data extraction was performed by two authors (SK and SB) and verified by three independent authors (SNS, SK1 and SD) as in previous studies [97,98] for accuracy, and discrepancies were resolved during the extraction process. No disagreements occurred during the verification of extracted data. When a study did not report sufficient data to compute an effect size (missing means, standard deviations, or convertible statistics), the study was excluded from the quantitative synthesis. Data extracted from these studies included study design, participant characteristics such as age, gender, methods used, key findings, and observations (Supplementary Table S1). Eligible studies were systematically reviewed to examine patterns in YVC technique duration and variations in study design and the primary outcome measures. Key methodological characteristics, including participant demographics (Figure 2a), intervention protocol, and assessment tools (Supplementary Table S2), were extracted to identify trends and variations across the literature, which were represented in Figure 2.

A comprehensive meta-analysis was conducted to explore the rationale for YVC-based interventions. A total of 34 studies were included, each selected based on methodological quality and relevance to the research objective. Among the included studies, 12 were randomized controlled trials, 7 were quasi-experimental designs, and 10 were pre–post intervention studies that examined the practice of YVC over time (Figure 2b,c). Three studies using a self-as-control approach were examined. Two repeated measures contributed to the dataset. The distribution of YVC-related parameters was documented for the session duration and had moderate variability, with a median of 25 min and an interquartile range of 15–40 min (Figure 2d). Training duration showed a distribution with a median of 4 weeks and an interquartile range of 2.25–8 weeks, indicating that most experiments were conducted for short to medium duration (Figure 2d). All study types were considered and weighted in the synthesis process, ensuring that methodological variations were addressed in the final analysis. A list of keywords was used to represent an overview of participant characteristics, health conditions, outcome domains, and reported effects related to the included YVC studies [99,100].

### 2.4. Evaluation of Methodological Bias in Included Studies

Cochrane risk of bias tool (RoB1), as described in the Cochrane Handbook (Version 5.1.0) and operationalized per [101,102], was applied uniformly across all included study designs to enable direct comparison within a single reporting framework. The Cochrane ROB 1 domain-by-domain assessment was applied only after eligibility screening to characterize the risk of bias profile of the retained studies. Domains specific to randomization were marked as not applicable for non-randomized or quasi-experimental studies rather than scored. The six standard ROB1 domains assessed were selection bias, performance bias, detection bias, attrition bias, reporting bias, and other sources of bias, each rated as low, high, or unclear risk. The risk of bias assessment was performed independently by two authors (SK and SB), and there were no disagreements in this study due to the limited literature on YVC.

### 2.5. Classification of Study Designs and Outcomes

All 34 eligible studies were included, and risk of bias was assessed. Five studies were excluded [2,51,66,103,104] from the meta-analysis because of insufficient statistical information for reliable effect size estimation. No effect sizes were imputed. For the quantitative analysis, 29 studies were included.

The included studies were classified and listed based on their outcomes and study designs in Table 2.

### 2.6. Statistical Analysis and Assessment of Heterogeneity

Statistical analyses were conducted to evaluate the effects of YVC-based outcomes on cognitive, visual, and psychophysiological domains, including within-subject (pre–post) and between-subject (YVC–control) study designs. Raw outcome data were extracted from the included studies and classified into the prespecified functional domains. Individual study-level Hedges’ g, standard errors, and 95% confidence intervals were calculated separately for within-subject and between-subject studies using design-specific formulas. When a study reported multiple outcomes, their corresponding outcome-level effect sizes were averaged into a single composite study-level Hedges’ g to preserve the study as the unit of analysis [105]. All the notations and parameters used are described in Supplementary Tables S3 and S4. All the parameters were calculated using jamovi (Version 26) [106] and PythonMeta (Version 1.26) [107].

Effect magnitudes across diverse outcomes were computed using the standardized mean difference (SMD). Effect sizes were calculated separately according to study design. For between-subject studies, Cohen’s d was calculated from the difference between group means and the pooled standard deviation:

(1) $$d=\frac{M_{2}-M_{1}}{{\mathrm{SD}}_{\mathrm{pooled}}}$$

where

(2) $${\mathrm{SD}}_{\mathrm{pooled}\;}=\sqrt{\frac{\left(n_{1}-1\right){\mathrm{SD}}_{1}^{2}+\left(n_{2}-1\right){\mathrm{SD}}_{2}^{2}}{n_{1}+n_{2}-2}}$$

Hedge’s g was obtained by applying the small-sample bias-correction factor:

*g* = *J* × *d* (3)

where

(4) $$J=1-\frac{3}{4\mathrm{df}-1}$$

For within-subject designs, dependent-measures effect sizes were calculated to account for the correlation between pre- and post-intervention measurements. When a paired-samples t statistic was reported, the standardized mean change was calculated as:

(5) $$d_{z}=\frac{t}{\surd n}$$

Followed by the Hedges’ correction:

*g* = *Jd_z_* (6)

The corresponding standard error was calculated as:

(7) $${\mathrm{SE}}_{g}=J\sqrt{\frac{1}{n}+\frac{d_{z}^{2}}{2\left(n-1\right)}}$$

When only pre- and post-intervention means and standard deviations were available, the SD of the change was calculated as:

(8) $$SD_{\Delta }=\sqrt{SD_{\mathrm{pre}}^{2}+SD_{\mathrm{post}}^{2}-2\mathrm{rS}D_{\mathrm{pre}}SD_{\mathrm{post}}}$$

where r represented the pre–post correlation. The pre–post correlation was not consistently reported in the studies; therefore, r = 0.50 was adopted as the primary working assumption [108,109], with r = 0.30 and r = 0.70 examined in sensitivity analyses. The standardized mean change (effect size d) was then calculated as:

(9) $$d=\frac{M_{\mathrm{post}}-M_{\mathrm{pre}}}{{\mathrm{SD}}_{∆}}$$

and converted to Hedge’s g using the correction factor *J* (as derived from Equations (3), (4) and (8)).

A random-effects model employing the inverse-variance method was used to compute the pooled effect sizes across studies. The weight, *w_i_* of the ith study was computed.

(10) $$w_{i}=\frac{1}{{\mathrm{SE}}_{i}^{2}+\tau^{2}}\;$$

The pooled effect size, $\hat{\mu }$ was calculated.

(11) $$\hat{\mu }=\frac{\sum w_{i}y_{i}}{\sum w_{i}}\;$$

The chi2 (χ^2^) test was used to assess variations between categorical variables, with statistical significance at *p* < 0.05.

(12) $$Q=\sum {w_{i}\;\left(y_{i}-\hat{\mu }\right)}^{2}\;$$

The Hartung–Knapp–Sidik–Jonkman (HKSJ) method was used to improve confidence interval coverage, particularly with few studies. HKSJ-adjusted standard errors and 95% CIs were calculated using random-effects inverse-variance weights and the corresponding degrees of freedom.

(13) $${\mathrm{SE}}_{\mathrm{HK}}=\sqrt{\frac{\sum {w_{i}\;\left(y_{i}-\hat{\mu }\right)}^{2}}{\left(k-1\right)\sum w_{i}}}$$

(14) $$\mathrm{CI}=\hat{\mu }\pm \;t_{k-1}\times {\mathrm{SE}}_{\mathrm{HK}}\;$$

Within-subject and between-subject random-effects meta-analyses were performed across each functional domain using Cochrane Review Manager (RevMan 5.4) [110], with inverse-variance weighting and REML estimation of between-study variance (τ^2^), with HKSJ-adjusted confidence intervals. To provide an overall descriptive summary of magnitude and direction of effects across functional domains, domain-level composite Hedges’g and standard error estimates were calculated using the arithmetic mean.

Heterogeneity was assessed using Chi-square (χ^2^), I^2^, and τ^2^, and pooled confidence intervals were calculated using the HKSJ method. Heterogeneity across the included studies was classified using the following thresholds: 0–24% (low), 25–49% (moderate), 50–74% (substantial), and 100% (considerable) [111].

For the meta-regression analysis, 9 studies that involved the cognitive domain were included to assess whether variations in intervention duration, measured in minutes, were associated with different outcomes across studies. This analysis reveals how extended practice durations may enhance intervention efficacy. A linear regression model was used to test their association, with the fitted line illustrating the trend linking session duration in minutes to domain outcome.

*Effect size* = *β*_1_ (*Duration*) + *β*_0_ (15)

Change in SMD across the individual study was calculated as β. The *p*-value tested the null hypothesis that β = 0 (no linear relationship between duration and effect size).

(16) $$\begin{array}{c} \beta =\frac{\sum \;\left[\left(x_{i}-\bar{x}\right)\left(y_{i}-\bar{y}\right)\right]}{\sum \left[{\left(x_{i}-\bar{x}\right)}^{2}\right]}\\ t=\frac{\beta }{\mathrm{SE}\left(\beta \right)}\; \end{array}$$

Positive SMD values were interpreted as indicating improvement in YVC outcomes compared to baseline conditions.

Effect sizes were interpreted using Cohen’s conventional thresholds: SMD = 0.2–0.4 (small), 0.5–0.7 (moderate), indicating improvement in the intervention compared to the control or baseline condition, but not uniform across all studies, and SMD ≥ 0.8 (large). Statistical significance was set at *p* < 0.05.

## 3. Results

### 3.1. Prevalence and Distribution of Systematic Keywords to Classify Outcome Domains in the YVC Research

**Figure 3:**
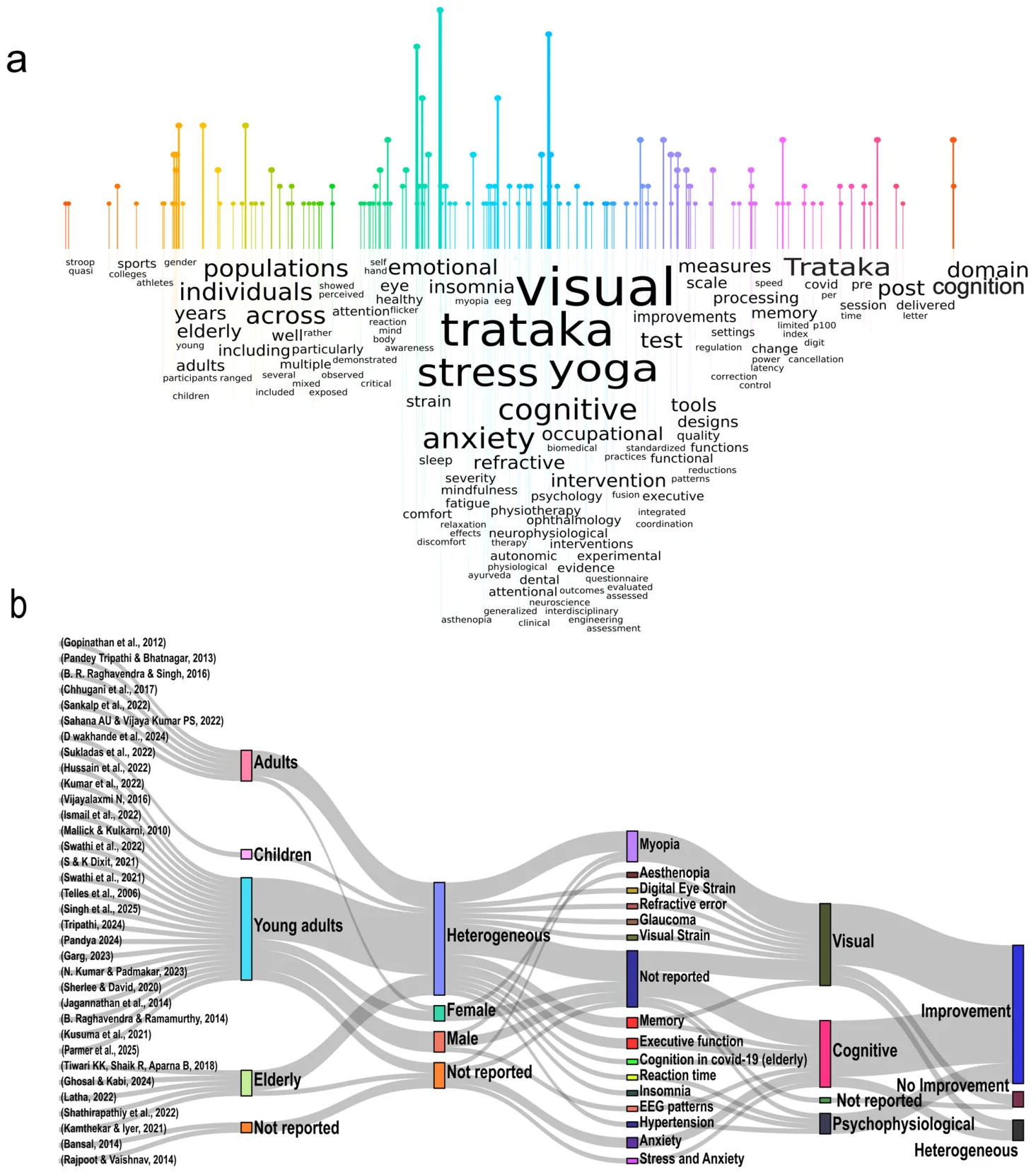
Key phrase relevance and heterogeneity in participant characteristics across included YVC studies [2,3,4,5,38,44,51,57,58,66,67,81,89,90,103,104,112,113,114,115,116,117,118,119,120,121,122,123,124,125,126,127,128,129]. (**a**) The word size plot represents Trataka-related term frequency, and spatial clustering signifies semantic relatedness, indicating visual, stress-related, and cognitive themes across included studies. The bigger the size of the word, the more often it was used, and the smaller the size, the less it was mentioned across the literature. (**b**) Sankey diagrams represent the YVC-based intervention studies, denoting the flow of data from individual studies to the characteristics of participants, reported health conditions, outcome domains related to functionality, and the effects of the intervention. The flow chart describes the contributions of studies by age group (children, young adults, elderly), gender distribution (male, female, heterogeneous), disease conditions (myopia, visual strain, anxiety, insomnia, post-COVID-19 disorders), and the main functional domains. The size of the flow in each category indicated the contribution of the studies, offering a collective assessment of characteristics of the population, the intervention, and the outcomes of the studies conducted on YVC.

Studies have shown varying levels of distribution, prominence, and co-occurrence across the literature for the obtained keywords related to various domains of neuroscience. These keywords have been clustered horizontally by relevance to YVC studies, and the word size indicates how often they appear in the literature, while their spatial proximity indicates how closely they are related (Figure 3a). The central words “Trataka”, “Visual”, “Stress”, “Yoga”, and “Cognitive” are the most often listed. The overlap between cognitive and visual clusters has led many YVC-based studies to conceptualize both visual training and cognitive performance as interrelated rather than independent domains. Methodological terms (including measures, tools, designs, functional, and intervention) remained more isolated, which indicated heterogeneity rather than a standardized approach.

Across the 34 studies, the thickness and flow of the ribbon model represented the distribution of studies with diverse populations across different age groups and genders, participants with distinct health conditions, and mixed outcomes after practicing the YVC (Figure 3b). These 34 studies have included participants from different age groups, from 8 to 11 years [103,104,112], 18 to 25 years [2,3,51,57,58,67,81,89,90,103,104,112,113,114,115,116,117,118,119], 25–40 years, and 40–80 years [4,5,38,44,66,120,121,122,123,124,125,126,127,128,129]. This suggests the diversity and predominance of young populations, while one study did not report the age group [107]. A gender-based heterogeneity was observed across the 34 studies, among which 10 studies labeled them as heterogeneous (mixed population) [2,51,103,104,107,113,121,124,125,126], and 7 studies specified males only or females only. Furthermore, six studies did not report any information about the involvement of participants [5,38,66,81,89,115]. Various vision-related conditions, especially myopia, digital eye strain, visual strain, asthenopia, and glaucoma, were reported in 15 studies [2,3,5,51,103,113,114,115,116,120,122,125,126]. Four studies included stress- and anxiety-related conditions, insomnia, and hypertension [4,124,128,129]. Cognitive improvements have been observed in visual-related studies after YVC practice [38,44,57,58,66,67,81,89,104,116,117,118,124,127]. Only six studies did not report any improvement or had heterogeneous results, which may indicate inconsistent intervention effects or differences in the methods of measuring the outcomes [103,109,113]. The existence of heterogeneous and non-reported data depicts differences in reporting practices and outcomes among the studies. These visualizations provide a descriptive and exploratory characterization of the included studies and participant characteristics and are not part of the quantitative statistical analysis and were not used to estimate intervention effects.

### 3.2. Non-Uniform Intervention Parameters and Associated RoB Across YVC Studies

The RoB evaluation indicated differences in methodological quality across studies, including eligibility criteria, search strategy, study selection, reporting transparency, and interpretation. RoB was assessed in the following domains: selection bias, performance bias, detection bias, reporting bias, attrition bias, and other sources of bias. The domain-specific assessment was applied to randomized studies, while the same domain structure was used to consider descriptively for non-randomized and quasi-experimental studies to facilitate consistent reporting across the evidence. Randomization-specific domains, including sequence generation and allocation of concealment, were marked as not applicable for studies without random allocation rather than being assigned to a risk category.

**Figure 4:**
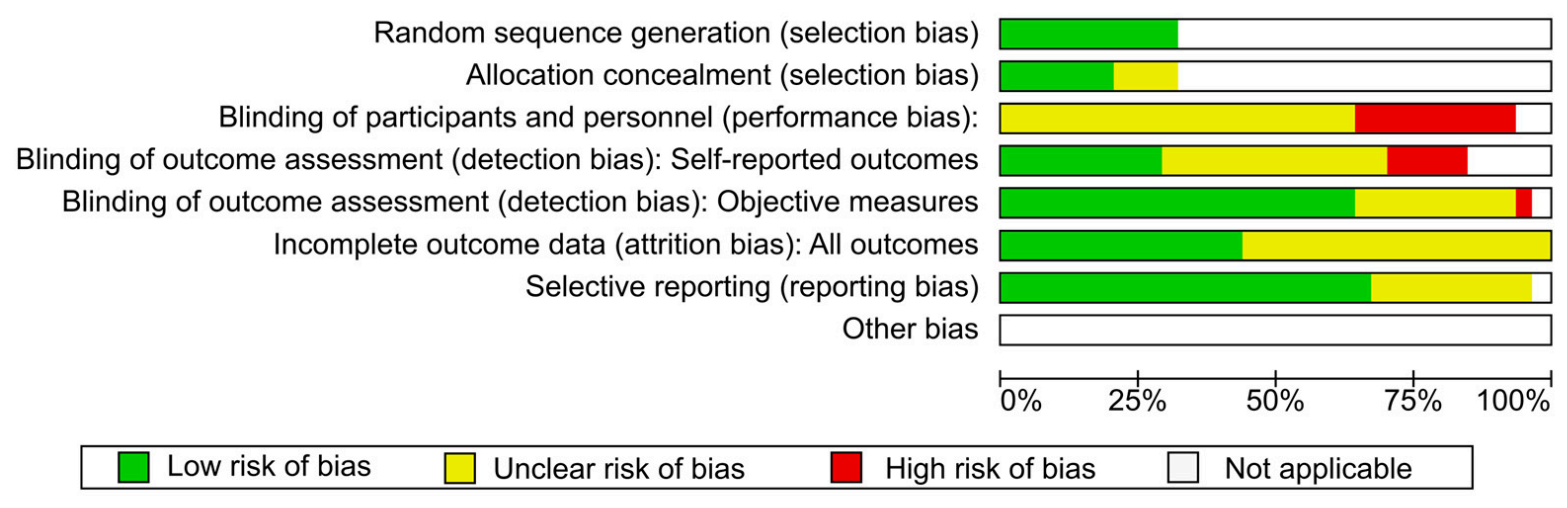
Risk of bias summary for the 34 included studies assessing YVC-based interventions (also see Supplementary Figure S1). The distribution of risk of bias across 34 studies was estimated using the Cochrane risk of bias assessment tool. A horizontal bar represented a domain of bias, and the colors showed the proportion of studies rated as low risk (green), unclear risk (yellow), and high risk (red), whereas the white space denoted insufficient information from the data to conclude the domain result.

RoB assessments indicated that the random sequence generation was rated as low risk in 11 studies (33%) (green). Allocation concealment was computed as low risk in seven studies (21%) and unclear in four studies (12%) (yellow). The remaining studies were not considered applicable as they involved no randomization or used quasi-experimental designs (Figure 4).

For blinding of participants and personnel, 21 studies (65%) were classified as unclear risk and 11 studies (32.4%) as high risk (red), reflecting the practical difficulty of blinding participants in YVC-based studies. For self-reported outcomes, approximately 9 studies (30%) were at low risk, 15 studies (41%) at unclear risk, and 5 studies (15%) at high risk. However, objective outcome assessment demonstrated a comparatively favorable profile: 22 studies (65%) were low risk, 11 studies (29%) were unclear, and 1 study (3%) was high risk. Incomplete outcome data were classified as approximately 15 studies (43%) low risk and 18 studies (57%) unclear, whereas selective reporting showed 22 studies (68%) low risk and 10 studies (29%) unclear. Other biases were not applied as a substantive risk domain in the assessment. Overall, the evidence base demonstrated a relatively lower risk for objective selective reporting and outcome assessment. The individual study for each domain, from selection to other sources of bias, was assessed separately to summarize this risk of bias graph (Supplementary Figure S1).

### 3.3. Cognitive Outcomes Across Short- and Long-Term YVC Practice Experimental Studies

**Figure 5:**
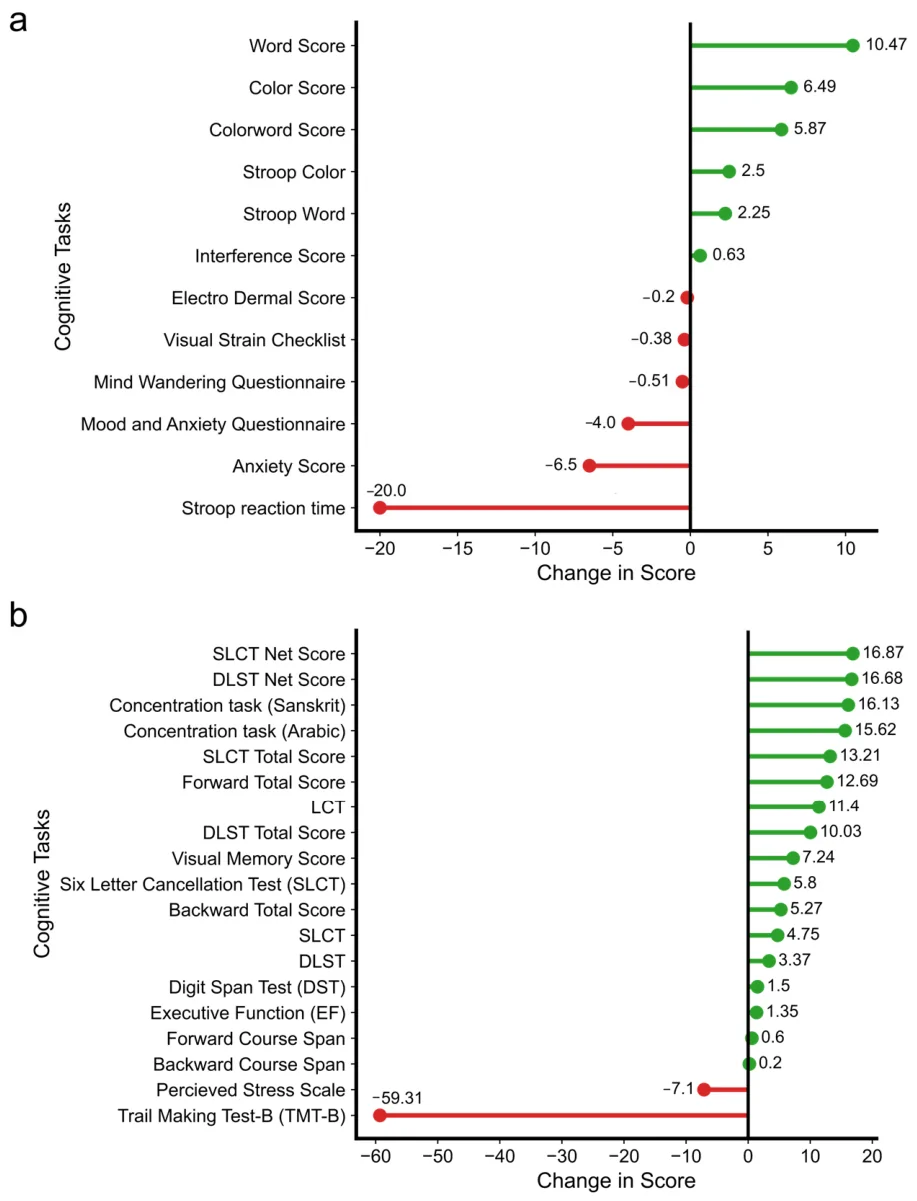
Task-based score analysis for cognitive, visual, and psychophysiological outcomes. Variations across task-based scores highlighted the significance of YVC-related effects across cognitive, visual, and psychophysiological outcomes between the YVC and control groups (**a**,**b**) and pre–post changes within the YVC group. Positive values represented multi-domain benefits following YVC practice. Negative values indicated either a reduction in measures, where lower scores denoted improvement (e.g., anxiety, reaction time), or a smaller change than the control condition [38,44,57,58,67,81,112,114,124,127].

Among the 29 studies, 10 primarily focused on the cognitive domain, including a minor psychophysiological task, and analyzed the total task-based score. The studies included a pre (before YVC practice) and post (after YVC practice) within-subject study design and a between-subject study design (YVC–control). The change in score for each cognitive task showed a consistent pattern of improvement after practicing YVC across the different cognitive domains. After following the YVC practice, the positive and negative differences in task scores across various cognitive measures, ranging from Stroop reaction time (−20.00) to the word score (10.47), indicate positive effects in the control and YVC groups (Figure 5a). Similarly, the pre- and post-YVC groups showed positive and negative scores across the various cognitive tasks from the Trail-Making Test-B (TMT-B) (−59.31) to the SLCT net score (16.87), indicating positive effects following YVC practice (Figure 5b). Psychophysiological measures such as electrodermal activity, anxiety (assessed by the Hamilton scale), Mood and Anxiety symptoms (MASQ), Stroop reaction time, perceived stress scale (PSS), and TMT-B showed negative scores, which indicate improvement because lower scores signify reduced anxiety and faster reaction time performance after YVC practice [38,44,57,58,67,81,112,114,124,127].

**Figure 6:**
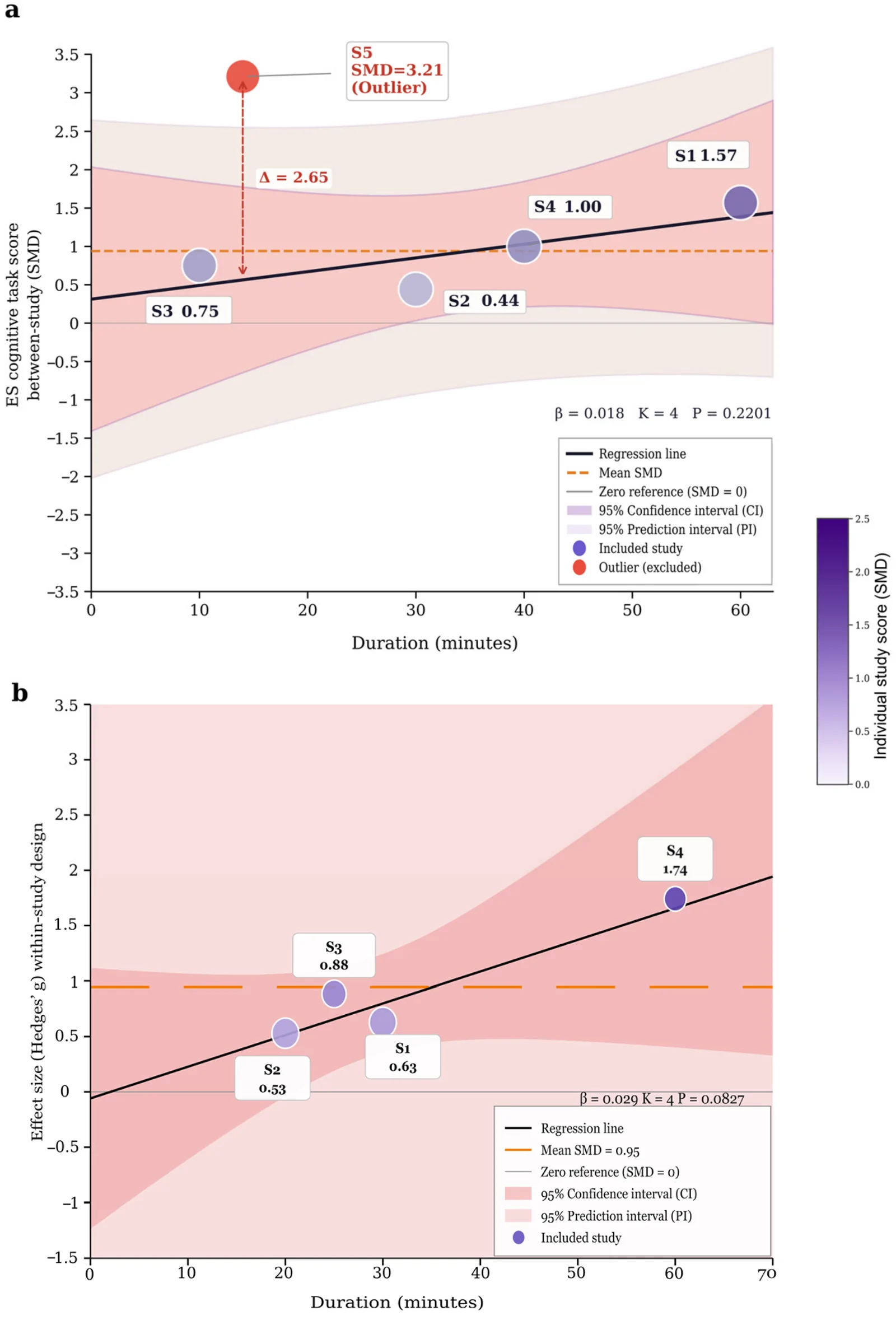
Meta-regression plots inspecting session duration as a moderator of effect size (SMD) across YVC-based intervention studies. (**a**) Four included studies; one outlier excluded, and (**b**) cognitive task outcomes within-subject study designs (K = 4 studies). Each bubble represented a single study, with color intensity indicating effect size, where dark shades represented larger SMD values. The solid black line denotes the ordinary least squares regression line fitted to the included studies. The dark shaded band indicates the 95% confidence interval (CI), where the plausible range of the true regression line lies, and the SE incorporates both sample size and distance from the mean duration. The light shaded band denotes the 95% prediction interval (PI), suggesting the expected range for future studies. The orange dotted line represents the slope, indicating the change in SMD per additional minute of session duration. In (**a**), duration was not a significant moderator overall (β = 0.018, *p* = 0.2201), with S5 identified as an outlier (∆ = 2.65 SMD units above prediction), pointing to a potential study-level factor that may influence the direction of the effect. In (**b**), duration has a positive but non-significant trend (β = 0.029, *p* = 0.083), due to K = 4 [3,38,44,57,58,81,112,116,127].

A meta-regression analysis was performed to identify whether improved cognitive task scores were associated with the duration of YVC practice sessions (10–60 min) as the indicator [3,38,44,57,58,81,112,116,127]. Across the nine studies, the results showed that as the duration of YVC practice increased, the effect size increased. Cognitive outcomes were converted to Hedge’s g, and multiple outcomes within the same study were summarized into a single study-level effect size. Five between-subjects studies were included in the regression analysis, where one study remained an outlier due to its observed effect size of 3.21, which fell beyond the 95% prediction interval of the regression model fit to the other four studies (SMD = 2.65), and exceeded the model’s predicted value at its corresponding session duration (Figure 6a). A sensitivity analysis was conducted (K = 5; see Supplementary Figure S5). Both including (β = 0.009, *p* = 0.7891, K = 5) and excluding the study (β = 0.018, *p* = 0.2201, K = 4) showed no significant association between session duration and effect size, indicating that duration alone may not be a strong moderator. The mean effect size across four studies was 0.94, and individual effect sizes ranged from 0.44 to 1.57.

Similarly, session duration did not show a statistically significant association with effect size in the within-subject cognitive studies (β = 0.029, *p* = 0.083, K = 4). The mean effect size across the four included studies was 0.946, while individual study effect sizes ranged from 0.530 to 1.741. All four study-level estimates were positive, with the lowest effect observed at 20 min (0.530) and the highest at 60 min (1.741) (Figure 6b). Although the trend shows larger effects with increasing duration, it was not statistically significant. No outliers were identified. Therefore, the current evidence from both between-subject and within-subject designs was insufficient to establish a duration–response relationship for cognitive outcomes. The wide confidence and prediction intervals indicate substantial uncertainty in the estimated relationship and may reflect factors beyond duration.

### 3.4. Pooled Effects of YVC Practice Favored Visual, Cognitive, and Psychophysiological Outcomes Across Within- and Between-Subject Study Designs

**Figure 7:**
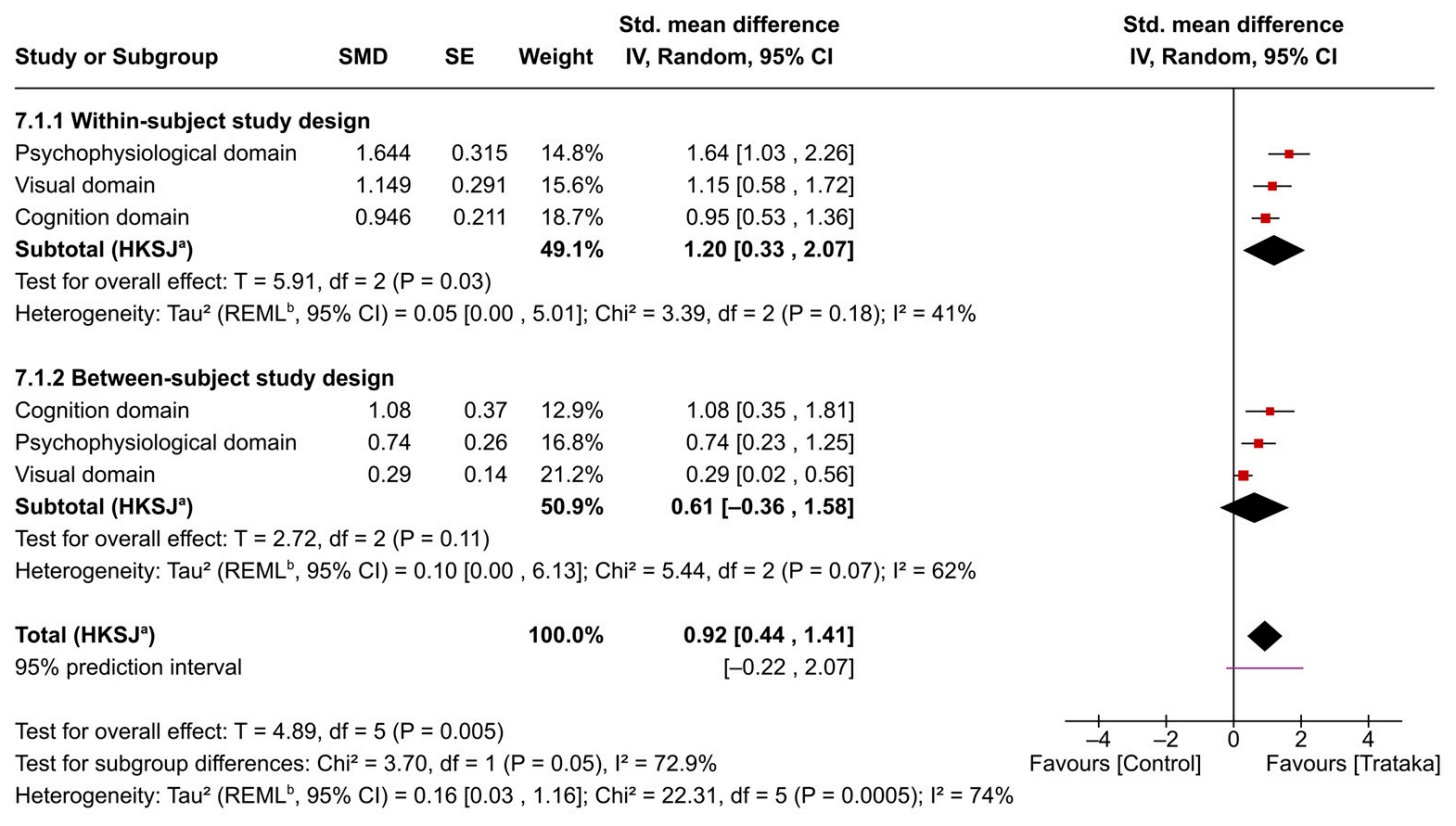
Effects on YVC practitioners across within- and between-subject studies on visual, cognitive, and psychophysiological domains. Pooled SMDs for within-subject and between-subject study designs across the functional domains. A random-effects model with HKSJ-adjusted 95% confidence intervals was used. Heterogeneity was assessed (SMD = 0.61, 95% CI: −0.36–1.58; *p* = 0.11), with moderate heterogeneity (I^2^ = 62%, χ^2^ = 5.44, *p* = 0.07).

Within-subject designs showed a statistically significant pooled effect favoring YVC (SMD = 1.20, 95% CI: 0.33–2.07, *p* = 0.03), with moderate heterogeneity (I^2^ = 41%; χ^2^ = 3.39, *p* = 0.18). Between-subject designs also favored YVC, but the pooled effect was not statistically significant (SMD = 0.61, 95% CI: −0.36–1.58; *p* = 0.11), with moderate heterogeneity (I^2^ = 62%, χ^2^ = 5.44, *p* = 0.07). The subgroup difference between within-subject and between-subject pooled estimates was borderline statistically significant (χ^2^ = 3.70, *p* = 0.05, I^2^ = 72.9%), with a larger pooled effect observed in the within-subject designs (Figure 7). Overall, the pooled estimates favored YVC in both study designs, although the magnitude and statistical significance differed between designs. The pooled analysis, stratified by study design, showed a significantly positive effect in the within-subject studies across all domains and a smaller pooled positive effect for between-subject studies, with moderate heterogeneity reported in Supplementary Figure S2a,b.

In the RCT-only analysis, the psychophysiological domain favored YVC with statistical significance (SMD = 0.68, 95% CI 0.04–1.32, I^2^ = 87%), whereas the cognitive effect was positive when Hedge’s g favored YVC over the baseline condition, but not statistically significant (SMD = 1.02, 95% CI 0.25–2.29, I^2^ = 90%). The vision domain showed no significant effect and substantial heterogeneity (SMD = 0.86, 95%, I^2^ = 99%). Overall, the pooled RCT estimate was positive with statistical insignificance (SMD = 0.62, 95% CI 0.03–1.26, I^2^ = 95%), with a wide predicted interval (−1.78 to 3.02) (Supplementary Figure S4). Leave-one-out sensitivity analysis showed that the pooled effects remained positive across both study designs. In within-subject analyses, the largest changes were observed after removal of three studies (Δ = −0.37, Δ = −0.36, Δ = 0.30), while in between-subject analyses, removal of one study produced the largest increase and decrease (Δ = 0.56 and Δ = −0.40) (Supplementary Table S6 and Figure S3).

## 4. Discussion

This review and meta-analysis synthesizes the evidence on Trataka (YVC) across visual, cognitive, and psychophysiological outcomes. The frequent key phrases across the 34 studies indicated the relevance of YVC in the critical domains, and the study characteristics have reported diversity across participants’ age, gender, assessments, and YVC outcomes (visual strain, hypertension, anxiety).

The RoB assessment of the reported studies highlights several methodological inconsistencies, including participant selection for study designs, detection of diverse outcomes in the same domain, reported participants’ performance, and self-reported, selective, or inadequate outcomes. These reported factors led to a highly biased evaluation of YVC-based interventions, underscoring the need for stronger research designs in future studies. Participant selection in the studies indicated that only the younger population limited the interpretation of YVC effects among the children and the elderly populations. Diverse outcomes were observed due to several assessments (word score, HRV, sleep quality, MASQ, TMT-B, SLCT, Stroop reaction time, HARS, and PSS) and variation in protocols (gazing duration, intervention length, and session frequency from single sessions to weeks or months). This diversity limits the comparability of individual participants’ performance to employ generalized standard tools for further research. These selective protocol-based variations on YVC have led to diverse research objectives (visual strain, cognitive and psychophysiological stress) and study designs (including pre–post, quasi-experimental, repeated measures, and limited RCTs), indicating heterogeneity across the studies and implying the need for specific RCTs and domain-focused reproducible protocols to minimize bias.

In the meta-regression analysis, no statistically significant duration–response relationship between session length and cognitive outcomes was reported. Even within brief sessions (10–30 min), effect sizes on cognitive-related measures were slightly favored, suggesting that the short- and long-term duration protocol may impact YVC’s efficiency across all domains. Sensitivity analysis in the between-subject meta-regression yielded no significant association between session duration and cognitive effect sizes to interpret duration–response relationships.

Although several studies showed effects in a favorable direction, the number of studies showing such effects was not taken as the primary basis. Some studies reported no significant change between baseline and YVC practitioners. The overall findings in relation to the magnitude of the pooled effect estimates, confidence and prediction intervals, and between-study variability suggested substantial heterogeneity observed across the studies. This variability was also evident across the study characteristics, including differences in participants, intervention duration, comparators, outcome measures, and study designs. These factors may contribute to variation in the observed effect sizes. The preliminary findings support a favorable overall direction of effect associated with YVC across the evaluated domains, although this favorability remained inconsistent. The findings from the RCT-only sensitivity analysis indicated that the pooled evidence showed a favorable effect of YVC, with a larger estimate among within-subject designs than between-subject designs. The potential benefit may rely on evidence from within-subject designs rather than the moderate effect provided by the RCTs. The difference in effect estimates between RCT-only and combined analyses highlights the influence of study design on the observed effect magnitude. Leave-one-out analysis showed that exclusion of individual studies did not reverse the direction of effects in either within- or between-subject designs.

Several methodological limitations affect the interpretability and generalizability of findings. Most research focused on young adults (18–25 years), with limited attention to older adults (25–40 years), the elderly (40–80 years), or children (8–11 years). A secondary concern is the imbalance of gender representation. Many studies limited their data to only male or female populations, which may influence the observed outcomes. Heterogeneity in the duration protocol further limits comparability. YVC session durations ranged from 2 min to 2 h, with frequencies from weekly to daily and total durations from 2 weeks to 3 months. This variability complicates the identification of optimal practice parameters. Many studies considered YVC alongside other practices, thereby limiting YVC efficiency among participants. Studies assessed a wide array of variables (e.g., flicker fusion thresholds, Stroop tasks, working memory, heart rate variability, and visual strain) using diverse instruments. Small sample sizes increased the risk of statistical errors, and many studies relied on subjective or self-reported outcomes rather than validated objective tools. Together, this contributes to the substantial heterogeneity observed in the quantitative synthesis and suggests that current evidence may not completely indicate the true efficacy of YVC. Standardized protocols defining session length, frequency, and duration are needed to improve consistency and clinical applicability. Substantial heterogeneity across the studies limited direct comparability and may have influenced the consistency of the observed results. A methodological limitation of this review was the reliance on the common RoB1 tool across studies with heterogeneous designs. This review was restricted to studies published in English or accompanied by a comprehensive English abstract, which may introduce language bias by excluding relevant evidence published in Hindi, Sanskrit, and other languages. Publication bias and selective reporting could not be formally assessed because the domain- and design-specific subgroups contained fewer than 10 studies, limiting the reliability of small-study effect tests.

To better understand the effects of YVC, yoga-related neuroscience research using physiological and neuroimaging methods is needed. Longitudinal EEG with functional connectivity analysis and cortical mapping studies may help characterize how repeated YVC practice modifies large-scale brain network organization over time. While further mechanistic and longitudinal studies are needed, the existing evidence supports YVC’s potential role in cognitive enhancement, attentional training, and emotional regulation. Though the heterogeneity remains unclear, the cumulative evidence supports YVC’s potential as a holistic practice for improving cognition, visual function, and psychophysiological balance. YVC demonstrated observable effects across visual, cognitive, and psychophysiological domains that remain to be fully explored. However, the current evidence lacks definitive conclusions regarding its effectiveness. Overall, analysis suggests potential benefits across selected outcomes but does not establish YVC as an effective technique across all domains, underscoring the need for standardized protocols, larger controlled trials, and neuroimaging-based investigations to establish clinical relevance. Future studies should include broader age ranges and conduct age-specific analyses. Standardizing YVC protocols, including duration, visual cue type, preparatory practices, and session design, may reduce heterogeneity and enhance comparability across studies.

## 5. Conclusions

The findings from this preliminary evidence suggest potentially beneficial effects across the domains of cognition, vision, and psychophysiology. However, substantial heterogeneity among the included studies, concerns regarding risk of bias, small samples, and variability in intervention protocols limit the reproducibility and generalizability of these findings. To better understand the benefits and limitations of YVC practice, future studies need to adopt rigorous trials with adequate sample sizes, standardized assessments, and follow-up evaluations to assess both short- and long-term effects. Development of a core outcome set for YVC would further improve consistency and comparability across studies. By addressing these methodological limitations, future investigations would be better positioned to generate reliable, reproducible evidence on the cognitive and neurophysiological benefits of YVC practice, thereby potentially aligning with the United Nations (UN) SDG 3.

## Figures and Tables

**Table 1:** Search strategy for the eight databases, including Boolean operators, search dates, and number of records retrieved.

Database	Search String	Search Period	Record Retrieved
PubMed	“Trataka” [Title/Abstract] OR “Yogic visual concentration” [Title/Abstract] OR “Yogic gazing” [Title/Abstract] AND (“Cognition” [Title/Abstract] OR “Visual attention” [Title/Abstract] OR “EEG” [Title/Abstract] OR “fMRI” [Title/Abstract] OR “Physiology” [Title/Abstract] OR “Neural mechanisms” [Title/Abstract] OR “Visual pathways” [Title/Abstract])	November 2010–December 2025	47
Scopus	TITLE-ABS-KEY ((“Trataka” OR “Yogic visual concentration” OR “Yogic gazing”) AND (“Cognition” OR “Visual attention” OR “EEG” OR “fMRI” OR “Physiology” OR “Neural mechanisms” OR “Visual pathways”))	November 2010–December 2025	59
Web of Science	TS = (“Trataka” OR “Yogic visual concentration” OR “Yogic gazing”) AND (“Cognition” OR “Visual attention” OR “EEG” OR “fMRI” OR “Physiology” OR “Neural mechanisms” OR “Visual pathways”)	November 2010–December 2025	13
Cochrane Library (CENTRAL)	#1 “Trataka” OR “Yogic visual concentration” OR “Yogic gazing” #2 “Cognition” OR “Visual attention” OR “EEG” OR “fMRI” OR “Physiology” OR “Neural mechanisms” OR “visual pathways” #3 #1 AND #2	2014–December 2025	38
DOAJ	(“Trataka” OR “Yogic visual concentration” OR “Yogic gazing”) AND (“Cognition” OR “Visual attention” OR “EEG” OR “fMRI” OR “Physiology” OR “Neural mechanisms” OR “Visual pathways”)	November 2010–December 2025	19
ProQuest	noft((“Trataka” OR “Yogic visual concentration” OR “Yogic gazing”) AND (“Cognition” OR “Visual attention” OR “EEG” OR “fMRI” OR “Physiology” OR “Neural mechanisms” OR “Visual pathways”))	November 2010–December 2025	27
JSTOR	(“Trataka” OR “Yogic visual concentration” OR “Yogic gazing”) AND (“Cognition” OR “Visual attention” OR “EEG” OR “fMRI” OR “Physiology” OR “Neural mechanisms” OR “Visual pathways”)	November 2010–December 2025	22
Google Scholar	(“Trataka” OR “Yogic visual concentration” OR “Yogic gazing”) AND (“Cognition” OR “Visual attention” OR “EEG” OR “fMRI” OR “Physiology” OR “Neural mechanisms” OR “Visual pathways”)	November 2010–December 2025	55
Total (after de-duplication)			280

**Table 2:** Study designs and outcome domains of the included literature.

Study-Design Category	Analytical Classification	Studies Included in Qualitative Analyses	Studies Included in Quantitative Analyses	Visual Domain	Cognitive Domain	Psychophysiological Domain
Randomized controlled trials (RCTs)	Between-subject study design	12	11	2	5	8
Quasi-experimental	Between-subject study design	7	4	3	1	2
Pre–post intervention	Within-subject study design	10	9	3	1	5
Self-as-control	Within-subject study design	3	3	0	1	2
Repeated measures	Within-subject study design	2	2	0	2	0

## Data Availability

No new data were created during this study. Data sharing does not apply to this article. The listed data are available in the manuscript and its Supplementary File.

## Supplementary Materials

The following supporting information can be downloaded at: https://www.mdpi.com/article/10.3390/healthcare14213624/s1, Supplementary Figure S1: Risk of bias analysis for individual study; Supplementary Figure S2: Between- and within-subject study analysis on visual, cognitive and psychophysiological domains; Supplementary Table S1: Characteristics of observations and symbol-based interpretation on YVC -based interventions; Supplementary Table S2: Functional domains and assessment tools/tests explored across YVC studies; Supplementary Table S3: Study-level outcomes; Supplementary Table S4: Notation and parameters used in statistical analyses; Supplementary Table S5: Grade Scale to assess the Certainty of Evidence; Supplementary Table S6: Leave-one-out (LOO) analysis table for between- and within -subject studies; Supplementary Figure S3: Leave-one-out (LOO) analysis Forest plot for between- and within-subject studies; Supplementary Figure S4: Only RCT–sensitivity analysis; Supplementary Figure S5: Bubble plot by including one study in between-subject study analysis. Table S7: Operational definitions for the usage of different key terms in the studies; Supplementary Table S8: PRISMA checklist.

## Abbreviations

YVC	Yogic Visual Concentration
PRISMA	Preferred Reporting Items for Systematic Reviews and Meta-Analyses
SMD	Standardized Mean Difference
RoB	Risk of Bias
RCT	Randomized controlled trial
SLCT	Six Letter Cancellation Test
DLST	Digit Letter Substitution Test
VFS	Visual Fatigue Scale
VEP	Visual Evoked Potential
MASQ	Mood and Anxiety Symptom Questionnaire
HRV	Heart Rate Variability
PQSI	Pittsburgh Sleep Quality Index
ISI	Insomnia Severity Index
PSS	Perceived Stress Scale
MWQ	Mind Wandering Questionnaire
SMASS	State Mindfulness Attention Awareness Scale
HARS	Hamilton Anxiety Rating Scale
