# Supplementary material for: Effect of Trataka on Visual, Cognitive, and Psychophysiological Outcomes: A Systematic Review and Meta-Analysis
Source: Healthcare (Basel). 2026 Oct 26;14(21):3624. doi: 10.3390/healthcare14213624 (PMC13762722; doi:10.3390/healthcare14213624)
Supplement: Supplementary file 1 [file healthcare-14-03624-s001.zip › healthcare-4528069-supplementary.pdf]

Systematic Review

Effect of Trataka on Visual, Cognitive, and Psychophysiological Outcomes: A Systematic Review and Meta-Analysis

Sowmiya Krishna [1], Sandeep Bodda [1], Sushma Naranappa Salethoor [2], Shyamasundaran Kulangara [3], Rammanohar Puthiyedath [4], Veena Nair [5] and Shyam Diwakar [1,6,*]

Supplementary Figure S1: Risk of bias analysis for individual study domain-by-domain [2-5,38,44,51,57,58,66,67,81,89,90, 103,104,112-129].

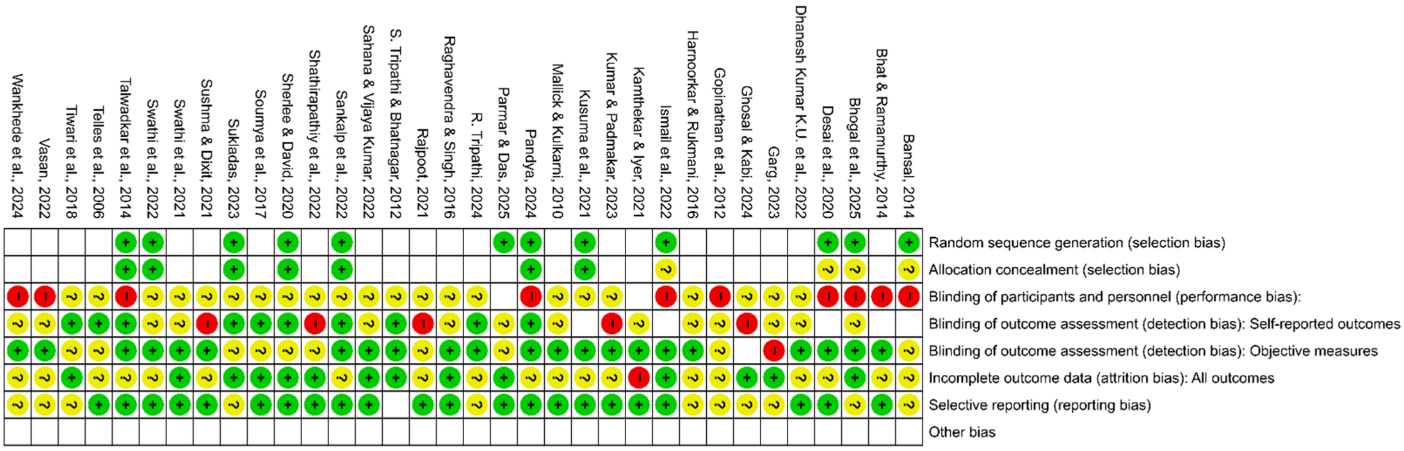

The Cochrane ROB 1 domain-by-domain assessment [98] was applied only after eligibility screening to characterize the risk-of-bias profile of the retained evidence base. The ROB 1 tool was applied uniformly across randomized and non-randomized study designs to enable direct comparison. Five domains were assessed across the 34 included studies. The assessment was based on selection bias, performance bias, detection bias, attrition bias, and reporting bias to determine the methodological quality of the individual study. Each horizontal bar represents one domain of bias, and the colors show the proportion of studies rated as low risk (green), unclear risk (yellow), and high risk (red), whereas the white space denotes not applicable based on the data for non-randomized studies to conclude the domain result.

Supplementary Figure S2:

Supplementary Fig S2 presents a separate, formal study-level random-effects meta-analysis (one composite effect size per study, inverse-variance weighted, with HKSJ-adjusted confidence intervals and REML-estimated τ^2) used specifically to quantify between-study heterogeneity and generate prediction intervals, which require a single independent effect estimate per study to be valid.

Between-subject study analysis on visual, cognitive and psychophysiological domains

The findings of 29 studies on Trataka suggested a positive effect on individual domains across visual, cognitive, and psychophysiological domains, favouring Trataka over controls. In the between-subject study design, Trataka showed a significant overall positive effect, with a pooled standardized mean difference (SMD) of 0.83(95% CI [0.33, 1.34], T= 3.44, df =

20, $P = 0.003$) under the random-effects model with the HKSJ method. This moderate-to-large effect indicates that Trataka practice is associated with positive improvements in these domains compared to control conditions, with the confidence interval excluding zero and the diamond positioned to the right of the null line (favouring Trataka). The wide 95% prediction interval [-1.39, 3.05] and very high overall heterogeneity ($I^2 = 94\%$, $\tau^2 = 1.08$ [95% CI 0.60, 2.52], $\chi^2 = 265.60$, df = 20, $P < 0.00001$) underscore the substantial heterogeneity due to different protocols, populations, and outcome measures. Individual domains, such as the visual domain (SMD = 0.37 (95% CI [-1.82, 2.57]), T = 0.4, df = 4, $P = 0.66$), demonstrated no statistical significance with high heterogeneity ($I^2 = 98\%$), evident. The cognition domain (SMD = 1.08 (95% CI [0.10,2.06]), T = 2.82, df = 5, $P = 0.04$), showed statistical significance with high heterogeneity ($I^2 = 86\%$), suggesting inconsistency due to differences in cognitive tasks. The psychophysiological domain pooled SMD was 0.85 (95% CI [0.32, 1.37], T = 3.64, df = 9, $P = 0.005$), indicating a moderately significant effect on psychophysiological parameters with substantial heterogeneity ($I^2 = 85\%$), suggesting variations in intervention adaptations.

Study or Subgroup	SMD	SE	Weight	Std. mean difference IV, Random, 95% CI
**3.1.1 Vision domain**				
Tiwari et al., 2018	-0.09	0.29	4.9%	-0.09 [-0.66 , 0.48]
Desai et al., 2020	-2.31	0.26	4.9%	-2.31 [-2.82 , -1.80]
Telles et al., 2006	2.47	0.25	4.9%	2.47 [1.98 , 2.96]
S. Tripathi & Bhatnagar, 2012	1.19	0.24	5.0%	1.19 [0.72 , 1.66]
Swathi et al., 2022	0.59	0.2	5.0%	0.59 [0.20 , 0.98]
**Subtotal (HKSJ[a])**			**24.7%**	**0.37 [-1.82 , 2.57]**
Test for overall effect: T = 0.47, df = 4 (P = 0.66)				
Heterogeneity: Tau² (REML[b], 95% CI) = 3.06 [1.06 , 25.79]; Chi² = 191.41, df = 4 (P < 0.00001); I² = 98%				
**3.1.2 Cognition domain**				
Parmar & Das, 2025	3.21	0.68	3.7%	3.21 [1.88 , 4.54]
Ghosal & Kabi, 2024	1.57	0.54	4.1%	1.57 [0.51 , 2.63]
Sukladas, 2023	1	0.38	4.6%	1.00 [0.26 , 1.74]
Talwadkar et al., 2014	0.43	0.27	4.9%	0.43 [-0.10 , 0.96]
Sherlee & David, 2020	0.48	0.22	5.0%	0.48 [0.05 , 0.91]
Pandya, 2024	0.75	0.19	5.1%	0.75 [0.38 , 1.12]
**Subtotal (HKSJ[a])**			**27.4%**	**1.08 [0.10 , 2.06]**
Test for overall effect: T = 2.82, df = 5 (P = 0.04)				
Heterogeneity: Tau² (REML[b], 95% CI) = 0.56 [0.11 , 6.30]; Chi² = 18.74, df = 5 (P = 0.002); I² = 86%				
**3.1.3 Psychophysiological domain**				
Parmar & Das, 2025	2.49	0.59	4.0%	2.49 [1.33 , 3.65]
Ghosal & Kabi, 2024	1.58	0.52	4.2%	1.58 [0.56 , 2.60]
Ismail et al., 2022	0.72	0.27	4.9%	0.72 [0.19 , 1.25]
Kusuma et al., 2021	0.25	0.26	4.9%	0.25 [-0.26 , 0.76]
S. Tripathi & Bhatnagar, 2012	1.39	0.26	4.9%	1.39 [0.88 , 1.90]
Sankalp et al., 2022	-0.39	0.24	5.0%	-0.39 [-0.86 , 0.08]
Bhogal et al., 2025	0.59	0.24	5.0%	0.59 [0.12 , 1.06]
Sherlee & David, 2020	1.1	0.24	5.0%	1.10 [0.63 , 1.57]
Swathi et al., 2022	0.5	0.2	5.0%	0.50 [0.11 , 0.89]
Pandya, 2024	1.12	0.2	5.0%	1.12 [0.73 , 1.51]
**Subtotal (HKSJ[a])**			**47.9%**	**0.85 [0.32 , 1.37]**
Test for overall effect: T = 3.64, df = 9 (P = 0.005)				
Heterogeneity: Tau² (REML[b], 95% CI) = 0.39 [0.15 , 1.91]; Chi² = 51.04, df = 9 (P < 0.00001); I² = 85%				
**Total (HKSJ[a])**			**100.0%**	**0.83 [0.33 , 1.34]**
95% prediction interval				[-1.39 , 3.05]

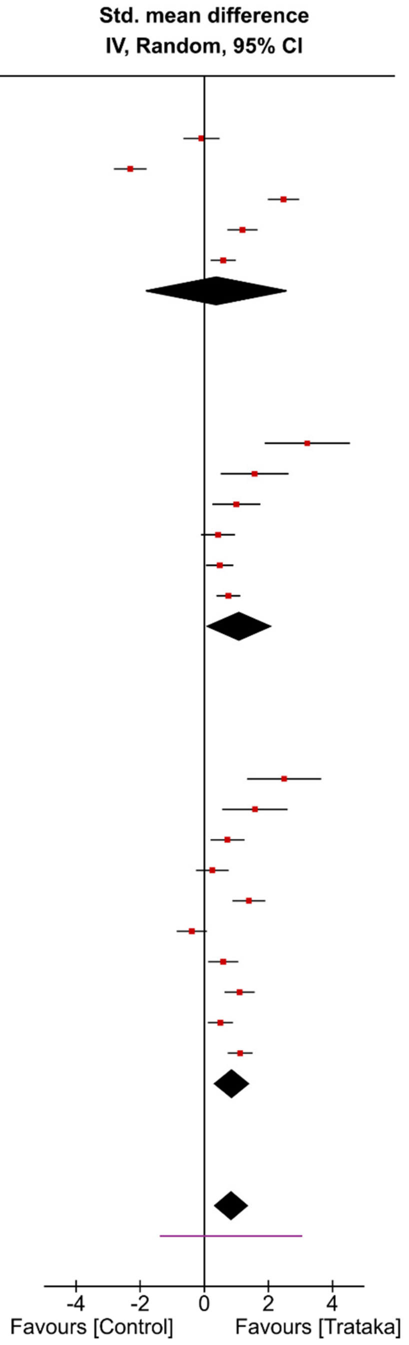

Test for overall effect: T = 3.44, df = 20 (P = 0.003)
Test for subgroup differences: Chi² = 0.70, df = 2 (P = 0.70), I² = 0%
Heterogeneity: Tau² (REML[b], 95% CI) = 1.08 [0.60 , 2.52]; Chi² = 265.60, df = 20 (P < 0.00001); I² = 94%

Footnotes
[a]CI calculated by Hartung-Knapp-Sidik-Jonkman method.
[b]Tau² calculated by Restricted Maximum-Likelihood method.

Supplementary Figure S2
a) Effect of YVC in between-subject study designs on visual, cognitive, and psychophysiological data [3, 67,81,112,113,114,116,117,119,121,123,124,125,127,129]

Within-study analysis on visual, cognitive and psychophysiological domains

Within-subject/pre–post analyses showed an overall pooled effect favouring Trataka (SMD = 1.26, 95% CI: 0.60–1.91; P = 0.001). The pooled effects were also statistically significant in the vision (SMD = 1.04, 95% CI: 0.03–2.05; P = 0.05), cognition (SMD = 0.90, 95% CI: 0.07–1.74; P = 0.04), and psychophysiological (SMD = 1.57, 95% CI: 0.09–3.05; P = 0.04) domains. Heterogeneity was moderate for vision (I^2 = 45%) and high for cognition (I^2 = 85%) and psychophysiological outcomes (I^2 = 98%), resulting in substantial overall heterogeneity (I^2 = 95%). The test for subgroup differences was not statistically significant (χ^2 = 1.03, df = 2, P = 0.60), indicating no clear evidence that effect magnitude differed significantly across functional domains. Although the overall pooled estimate favoured Trataka, the wide 95%

prediction interval (−1.08 to 3.59) indicates considerable uncertainty in the expected effect across future studies.

Study or Subgroup	SMD	SE	Weight	Std. mean difference IV, Random, 95% CI
**4.1.1 Vision domain**				
Soumya et al., 2017	1.677	0.4	6.7%	1.68 [0.89 , 2.46]
Wankhede et al., 2024	0.968	0.26	7.3%	0.97 [0.46 , 1.48]
Dhanesh Kumar K.U. et al., 2022	0.801	0.2	7.4%	0.80 [0.41 , 1.19]
**Subtotal (HKSJ[a])**			**21.4%**	**1.04 [0.03 , 2.05]**
Test for overall effect: T = 4.43, df = 2 (P = 0.05)				
Heterogeneity: Tau² (REML[b], 95% CI) = 0.06 [0.00 , 8.42]; Chi² = 3.84, df = 2 (P = 0.15); I² = 45%				
**4.1.2 Cognition domain**				
Sahana & Vijaya Kumar, 2022	1.741	0.293	7.1%	1.74 [1.17 , 2.32]
Raghavendra & Singh, 2016	0.884	0.2	7.4%	0.88 [0.49 , 1.28]
Garg, 2023	0.628	0.165	7.5%	0.63 [0.30 , 0.95]
Swathi et al., 2021	0.53	0.165	7.5%	0.53 [0.21 , 0.85]
**Subtotal (HKSJ[a])**			**29.6%**	**0.90 [0.07 , 1.74]**
Test for overall effect: T = 3.44, df = 3 (P = 0.04)				
Heterogeneity: Tau² (REML[b], 95% CI) = 0.21 [0.03 , 4.15]; Chi² = 14.16, df = 3 (P = 0.003); I² = 85%				
**4.1.3 Psychophysiological domain**				
Kumar & Padmakar, 2023	4.05	0.69	5.3%	4.05 [2.70 , 5.40]
R. Tripathi, 2024	3.66	0.39	6.8%	3.66 [2.90 , 4.42]
Shathirapathiy et al., 2022	1.76	0.29	7.1%	1.76 [1.19 , 2.33]
Rajpoot, 2021	1.14	0.23	7.3%	1.14 [0.69 , 1.59]
Bhat & Ramamurthy, 2014	0.6	0.19	7.5%	0.60 [0.23 , 0.97]
Sushma & Dixit, 2021	-0.17	0.19	7.5%	-0.17 [-0.54 , 0.20]
Mallick & Kulkarni, 2010	0.44	0.18	7.5%	0.44 [0.09 , 0.79]
**Subtotal (HKSJ[a])**			**49.0%**	**1.57 [0.09 , 3.05]**
Test for overall effect: T = 2.60, df = 6 (P = 0.04)				
Heterogeneity: Tau² (REML[b], 95% CI) = 2.35 [0.90 , 12.67]; Chi² = 120.28, df = 6 (P < 0.00001); I² = 98%				
**Total (HKSJ[a])**			**100.0%**	**1.26 [0.60 , 1.91]**
95% prediction interval				[-1.08 , 3.59]
Test for overall effect: T = 4.16, df = 13 (P = 0.001)				
Test for subgroup differences: Chi² = 1.03, df = 2 (P = 0.60), I² = 0%				
Heterogeneity: Tau² (REML[b], 95% CI) = 1.08 [0.54 , 3.49]; Chi² = 139.68, df = 13 (P < 0.00001); I² = 95%				

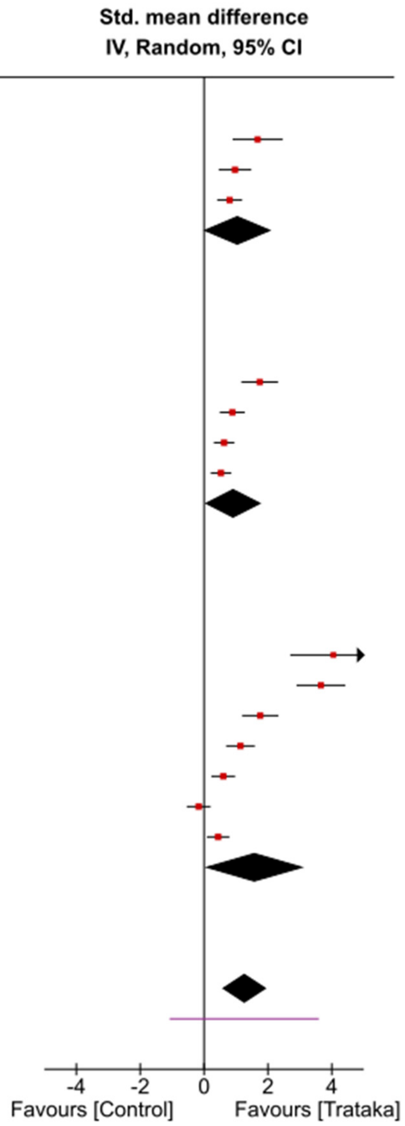

Supplementary Figure S2
b) Effect of YVC in within-subject study designs on visual, cognitive, and psychophysiological data [4,5,38,44,57,58,89,90,115,117,118,120,126,128]
(a and b) Pooled standardized mean differences with 95% confidence intervals from random-effects meta-analysis (HKSJ) of Trataka practice effects on visual, cognitive, and psychophysiological domains, stratified by between-subject and within-subject study designs. Each domain and pooled results indicated heterogeneity, assessed using REML-estimated τ² and I² statistics, with P values < 0.00001 for both between-subject and within-subject study designs.

Supplementary Table S1 Characteristics of observations and symbol-based interpretation on Trataka-based interventions

Ref/Study ID	Age	Gender (M/F)	Total	Comparator and follow - up duration	Study design and Domain	Intervention (session and duration)	Observation	Interpretation	Effect
[128]/S01	18–35	15/14	29	N/A and None	Pre–post/psychophysiological	10 days; 45 min/day	ISI decreased from 17.90 to 8.17 (p<0.001); PSQI global score and most sleep subscales also improved significantly; sleep-medication score was not significant	Suggests reduced insomnia severity and improved subjective sleep quality, potentially through down-regulation of cognitive/physiological arousal; uncontrolled design limits causal inference.	↑
[57]/S02	21–25	8/33	41	N/A and None	Repeated measures/ vision	2 weeks; 20 min/day	Forward Corsi span/total improved (p<0.001); backward span/total improved (p<0.05); Trataka outperformed baseline and eye-exercise session.	Supports enhancement of working memory, spatial memory and spatial attention, with eye-exercise comparison helping distinguish the effect from eye movements alone.	↑
[124]/S03	35–50	Mixed	104	Wait-listed control and None	RCT/psychophysiological	3 months; 8 min/session	Perceived stress (p=0.003), anxiety (p=0.006), parental/occupational stress (p<0.0001) decreased; self-perception and well-being improved (p<0.0001). Sleep quality was not significant within-group (p=0.054).	Suggests reduced psychological stress and improved mental well-being, with limited evidence for sleep improvement.	↓
[81]/S04	15–19	NR	20	No intervention control group and None	RCT/psychophysiological	4 weeks; 15 min/day	PSS and Letter Cancellation Test scores showed significant improvement after 4 weeks	Suggests simultaneous reduction in perceived psychological strain and improvement in sustained/selective attention, consistent with improved attentional regulation	↓↑
[44]/S05	24–75	22/40	62	N/A and None	Repeated measures/cognition	2 weeks; 30 min/session	MWQ decreased (p<0.001) and VSC decreased (p=0.002) in experimental group; between-group VSC p=0.035, but MWQ	Provides stronger evidence for reduced visual strain than for concentration; within-group mind-wandering reduction did not reach	↓↑

							p=0.072.	between-group significance	
[112]/S 06	8–11	Mixed	120	No intervention control group and None	RCT/ psychophysi ological	4 weeks; 2–10 min	MASQ anxiety decreased more with Trataka than relaxation ($p<0.001$); EDA during Stroop was lower ($p<0.01$); anxiety-related behaviours also reduced ($p<0.001$)	Indicates isreduced psychological and physiological arousal, with concomitant faster cognitive processing; EDA supports reduced sympathetic arousal.	↓
[118]/S 07	20–30	NR	30	N/A and None	Pre–post/ psychophysi ological	4 weeks; 20 min/day	Anxiety scores decreased significantly after Trataka	Suggests reduction in subjective anxiety/arousal, potentially reflecting improved relaxation and attentional regulation; absence of control limits attribution to Trataka	↓
[117]/S 08	15–21	100/0	100	N/A and None	Pre–post/ psychophysi ological	8 weeks; 15 min/day	Anxiety decreased significantly in both groups ($t=23.19$ and 14.10, $p<0.01$); concentration and retrieval tasks also improved ($p<0.01$)	Indicates concurrent reduction in anxiety and improvement in sustained concentration/retrieval, suggesting enhanced attentional stability alongside reduced psychological arousal; absence of control limits causal attribution	↑
[90]/S0 9	18–25	17/8	25	N/A and None	Pre–post/ psychophysi ological	3 weeks; 30 min/day	P100 latency did not change significantly after 3 weeks (left $p=0.497$; right $p=0.055$)	Indicates no measurable change in early visual-pathway conduction over 3 weeks; cognitive/attentional effects should not be interpreted as faster P100 transmission	↔
[113]/S 10	18–25	Mixed	24	Active comparator and None	Quasi-experimental /visual	8 weeks; NR	No significant improvement in refractive error or visual acuity after 8 weeks; intergroup $p>0.05$	Indicates Trataka did not produce measurable correction of refractive error or acuity over 8 weeks	×
[115]/S 11	15–25	NR	30	N/A and None	Pre–post/vision	2 weeks; 10 min/day	Vision did not improve significantly; asthenopia decreased significantly in Trataka group ($p<0.05$), while control showed no significant change	Suggests reduction in functional visual fatigue/asthenopia without measurable change in visual acuity/refractive status	↓
[2]/S12	15–25	Mixed	30	Active comparator and None	Quasi-experimental /vision	8 weeks; 15 min/day	Combined Yoga + Ayurveda group showed the best response in distant vision compared with Ayurveda alone and Trataka	Suggests any visual benefit was greater with combined intervention; isolated Trataka effect appeared weaker and	↑

							alone	cannot establish refractive correction	
[5]/S13	25–40	15/NR	30	N/A and None	Self-as-control/psychophysiological	Single session; 30 min/session	CFF increased significantly from 37.35±2.84 to 38.66±2.91 Hz after Trataka; control showed no significant change	Indicates acute enhancement of temporal visual-processing capacity, potentially reflecting altered cortical processing of visual stimuli rather than refractive change	↑
[104]/S14	10–19	Mixed	59	No intervention control group and None	Quasi-experimental/vision	3 weeks; online 25 min	Visual Stress Survey decreased significantly ($p<0.003$) and positive STA sentiments increased ($p<0.02$); concentration measures were not significant; control showed no significant changes	Suggests reduced visual strain and anxiety-related distress, but not demonstrable improvement in concentration over 3 weeks; effects cannot be attributed to Trataka alone	↓
[127]/S15	60–80	16/44	60	Wait-list control group and None	RCT/cognition	4 weeks; 30 min/day	SLCT and TMT-B improved immediately and at 1 month ($p<0.01$); digit span also improved at 1 month ($p<0.01$)	Suggests enhancement of attention, working memory and executive function	↑
[116]/S16	23	0/20	20	No intervention control group and None	Quasi-experimental/psychophysiological	8 weeks; 35–60 min/session	Simple reaction time, visual memory, hand-eye coordination and depth perception differed significantly at $p<0.05$; executive function was not significant	Suggests improvement in selected visuomotor and psychomotor processing rather than generalized executive function	↑
[114]/S17	20–23	21/85	106	No intervention control group and None	RCT/cognition	2 weeks; 20 min/day	VFS, VSC and mind-wandering decreased and mindfulness increased significantly after 2 weeks; between-group differences were significant	Suggests improved visual comfort and attentional regulation following Trataka	↔
[38]/S18	60–80	NR	30	N/A and None	Pre–post/cognition	4 weeks; 1 h/day	SLCT and DLST total/net scores increased and wrong responses decreased significantly after 30 days (all $p=0.000$)	Indicates improved selective/focused attention, visual scanning and psychomotor information processing; absence of control limits causal attribution	↑
[58]/S19	18–31	30/0	30	N/A and None	Self-as-control/cognition	2 weeks; 25 min/day	Stroop colour–word performance improved significantly after Trataka vs control ($p<0.001$).	Demonstrates a shift toward greater vagal/parasympathetic modulation and reduced autonomic arousal	↑
[67]/S2	16–19	42/38	80	Active control	RCT/psychop	NR	Stroop color, word and inference	Indicates enhanced selective	↑

0				and None	hysiological		scores improved significantly vs control (p<0.0001)	attention and cognitive processing.	
[103]/S 21	8–30	Mixed	60	Active control and 1-month	RCT/vision	12 weeks; 10 min/day	No significant improvement in visual acuity or clinical refraction; Yoga group showed greater reduction in pain, watering, eye strain and heaviness (p<0.005), while headache improved similarly in both groups	Suggests reduction of associated asthenopic symptoms rather than refractive correction; effects cannot be attributed specifically to Trataka because the Yoga protocol was combined	±
[3]S22	18–25	30/0	30	No intervention control group and None	RCT/cognitio n	8 weeks; 40 min/session	Significant post-test and adjusted post-test differences were reported after 8 weeks	Suggests enhanced visuo-motor processing efficiency, potentially through improved visual attention and speed of information processing	↑
[119]/S 23	18–25	16/80	96	No intervention control group and None	RCT/vision	8 weeks; 2 h/day	Refractive power showed a small reduction after 4 weeks, but not significant (right eye p=0.07; left eye p=0.11)	Limited short-term effect on refractive status; insufficient evidence for significant improvement	↑
[120]/S 24	18–40	Mixed	20	N/A and None	Pre–post/vision	8 weeks; 3×/week	CVS-Q decreased significantly (p<0.004) and Schirmer score changed significantly (p<0.001)	Suggests reduced subjective digital-eye-strain symptoms and altered tear-test response; uncontrolled design limits causal/mechanistic inference.	↓
[51]/S2 5	16–30	Mixed	66	Active control and 1-month	Quasi-experimental /vision	3 weeks; morning/evening	Trataka produced significant subjective relief for headache and watering in hypermetropia (p<0.01), but most objective refractive/ocular measures were not significant; 78.12% showed no objective improvement	Trataka produced significant subjective relief for headache and watering in hypermetropia (p<0.01), but most objective refractive/ocular measures were not significant; 78.12% showed no objective improvement	±
[121]/S 26	52–67	25/35	60	Active control and None	RCT/psychop hysiological	4 weeks; 55 min/day	SBP, DBP, RR, pulse, FBG and bilateral IOP decreased significantly after 1 month (p<0.05); control showed no significant change; all between-group comparisons p<0.05.	Indicates coordinated modulation of autonomic cardiovascular activity and ocular pressure, with accompanying glycaemic change; concurrent cardiovascular and IOP responses suggest systemic autonomic involvement.	↑
[122]/S 27	20–57	19/11	30	No intervention control group	Quasi-experimental /vision	13 weeks; 1 h/day	Visual discomfort and related visual symptoms decreased after the yoga intervention	Suggests reduced visual strain, possibly through relaxation and improved ocular regulation; effects	↓

				and None				cannot be attributed specifically to Trataka	
[123]/S 28	21–49	NR	291	Waitlist control group and None	Quasi-experimental /vision	8 weeks; 60 min/session	Visual discomfort decreased significantly after 60 days (p<0.001), while it increased in wait-list controls (p<0.001); group×time interaction p<0.001	Indicates reduced subjective visual fatigue/discomfort associated with regular yoga practice; intervention was not isolated Trataka	↓
[125]/S 29	18–60	Mixed	72	Active control and None	RCT/psychop hysiological	2 weeks; 30 min/session	Right-eye IOP decreased within intervention at day 14 (p=0.006) and day 28 (p=0.001), but between-group IOP differences were not significant	Suggests a possible ocular-pressure-lowering effect, but absence of significant between-group differences limits attribution to Trataka	±
[126]/S 30	25–35	Mixed	20	N/A and None	Pre–post/vision	8 weeks; 5 days/week	Foreign-body sensation, burning, discharge and redness decreased significantly (p≤0.0143); Schirmer test increased in both eyes (p=0.0052 and 0.0090); CVS-Q decreased from 13.38 to 9.45 (p<0.0002)	Suggests reduction in digital-eye-strain symptoms with improved tear-related ocular-surface parameters; effects cannot be attributed specifically to Trataka because Nasya was co-administered. Trataka reduces visual discomfort	↓
[89]/S3 1	NR	NR	40	N/A and None	Pre–post/ psychophysi ological	6 weeks; 10 min/day	Shooting performance increased markedly after 6 weeks; adjusted between-group difference was significant (F=191.84, p<0.001).	Indicates enhanced precision-based visuomotor performance, plausibly reflecting improved visual attention/attentional stability relevant to target shooting	↑
[4]/S32	20–33	30/0	30	N/A and None	Self-as-control/psych ophysiologic al	NR; 25 min/day	LF decreased (p<0.01), HF increased (p<0.01), HR decreased (p<0.01) and respiratory rate decreased (p<0.001) after Trataka; control showed no change	Demonstrates a shift toward greater vagal/parasympathetic modulation and reduced autonomic arousal	↑
[129]/S 33	25–45	46/14	60	No intervention control group and None	RCT/psychop hysiological	NR; 30 min/day	SBP, HR and mean RR decreased significantly immediately after Trataka (p<0.05); no significant changes in control	Suggests acute cardiovascular relaxation with possible autonomic/parasympathetic modulation	↓
[66]/S3 4	23–33	NR	6	N/A and None	EEG study/ psychophysiolo gical	NR; 4 min/session	EEG signals showed reduced irregularity and greater regulation during short-term Trataka	Suggests reduced neural-signal irregularity and a more regulated brain state during Trataka, interpreted as reduced stress/arousal	↓

Symbols: ↑ improvement; ↓ reduction; ↔ no significant change; ± mixed/moderate effect; × no effect.

VEP – Visual evoked potential; EEG-Electroencephalogram; NR-Not reported; RCT-Randomized controlled trial; CFF-Critical flicker fusion; ISI-Insomnia severity index; PSQI-Pittsburgh Sleep Quality Index; CBTT-Corsi block tapping task, IOP-Intraocular pressure; VFS- Visual Fatigue Scale, BP-Blood pressure; SBP-Systolic BP; DBP-Diastolic BP; RR-Respiratory rate; PR-Pulse rate; FBG-Fasting blood glucose; RE-Right eye; LE-Left eye; QoL-Quality of life. N/A – Within-subject designs; pre-intervention baseline serves as comparator; no separate control group. None – Outcomes assessed at a single post-intervention endpoint only; there was no subsequent follow-up assessment.

Supplementary Table S2 Functional domains and assessment tools/tests explored across Trataka studies

Functional domains	Assessment tools/tests	References
Sleep/sleep quality	Pittsburgh Sleep Quality Index (PSQI)	[124,128]
Sleep/sleep quality	Insomnia Severity Index (ISI)	[128]
Stress/anxiety/emotional well-being	Perceived Stress Scale (PSS)	[81,124]
Stress/anxiety/emotional well-being	Generalized Anxiety Disorder-7 (GAD-7)	[124]
Stress/anxiety/emotional well-being	Mood and Anxiety Symptom Questionnaire (MASQ) – Anxiety scale	[112]
Stress/anxiety/emotional well-being	Hamilton Anxiety Scale	[67]
Stress/anxiety/emotional well-being	Sinha's Comprehensive Anxiety Test	[118]
Stress/anxiety/emotional well-being	State Mindfulness Attention Awareness Scale (SMAAS)	[114]
Stress/anxiety/emotional well-being	Mind Wandering Questionnaire (MWQ)	[44,114]
Stress/anxiety/emotional well-being	Self-perception / well-being scales	[124]
Stress/anxiety/emotional well-being	Parental Stress Scale	[124]
Stress/anxiety/emotional well-being	Occupational Stress Index	[124]
Stress/anxiety/emotional well-being	Observed anxiety-related behaviours	[112]
Psychophysiological/autonomic	Electrodermal Activity (EDA)	[112]
Psychophysiological/autonomic	Heart Rate Variability (HRV: LF, HF, LF/HF)	[4]
Psychophysiological/autonomic	Heart rate / pulse rate	[4,129]
Psychophysiological/autonomic	Respiratory rate	[4,129]
Psychophysiological/autonomic	Systolic and diastolic blood pressure	[129]
Psychophysiological/autonomic	Fasting blood glucose	[121]
Psychophysiological/autonomic	Electroencephalography (EEG)	[66]
Visual/ocular	Snellen visual acuity chart	[2,51,103,113,115,119]
Visual/ocular	Retinoscopy / clinical refraction	[51,103,113,119]
Visual/ocular	Autorefractometer	[51]

Visual/ocular	A-scan / ocular biometry	[51]
Visual/ocular	Keratometer	[51]
Visual/ocular	Intraocular pressure (IOP) measurement	[121,125]
Visual/ocular	Visual Evoked Potential (VEP; P100 latency)	[90]
Visual/ocular	Critical Flicker Fusion (CFF)	[5]
Visual/ocular	Visual Fatigue Scale (VFS)	[114]
Visual/ocular	Visual Symptoms Checklist (VSC)	[44,114]
Visual/ocular	Visual discomfort questionnaire	[117,123]
Visual/ocular	Computer Vision Syndrome Questionnaire (CVS-Q)	[120,126]
Visual/ocular	Schirmer test	[120,126]
Visual/ocular	Asthenopia / ocular symptom assessment	[115,126]
Visual/ocular	Digit Span Test	[127]
Visual/ocular	Six Letter Cancellation Test (SLCT)	[38,44,127]
Cognition/attention/memory	Digit Letter Substitution Test (DLST)	[38]
Cognition/attention/memory	Trail Making Test-B (TMT-B)	[127]
Cognition/attention/memory	Stroop Color–Word Test	[58,67,112]
Cognition/attention/memory	Corsi Block-Tapping Test (CBTT)	[57]
Cognition/attention/memory	Letter Cancellation Test (LCT)	[81]
Cognition/attention/memory	Concentration / retrieval task	[117]
Psychomotor/perceptual performance	Simple Reaction Time (SRT) – Human Benchmark	[116]
Psychomotor/perceptual performance	Visual Memory – Human Benchmark	[116]
Psychomotor/perceptual performance	Hand–Eye Coordination – CogniFit	[116]
Psychomotor/perceptual performance	Depth Perception – CogniFit	[116]
Psychomotor/perceptual performance	Executive Function – CogniFit	[116]
Psychomotor/perceptual performance	Shooting performance / shooting score	[89]

Supplementary Table S3 Study-level outcomes

Outcome direction was retained according to the original measurement scale and was consistently applied in the calculation and presentation of effect sizes in the forest plots. Where lower scores represented a favourable outcome (e.g., anxiety, visual discomfort, symptom severity, or latency measures), reductions were interpreted as favourable changes. No arbitrary sign reversal was applied solely to obtain positive effect sizes. The original outcome direction and the direction used for interpretation.

a) **Between-study design: Individual outcome effect sizes (Hedges' g)**

Outcome	Domain	Hedges' g	SE	95% CI Lower	95% CI Upper
Far Vision (R)	Visual	0.98	0.203	0.58	1.38
Far Vision (L)	Visual	1.40	0.206	1.00	1.80
Near Vision (R)	Visual	1.35	0.25	0.86	1.84
Near Vision (L)	Visual	1.03	0.023	0.99	1.08
Dry Eye Questionnaire	Visual	2.47	0.25	1.98	2.96
Snellen's chart & autorefraction Equipment - Refractive Error (R)	Visual	−0.09	0.29	−0.66	0.48
Snellen's chart & autorefraction Equipment - Refractive Error (L)	Visual	−0.01	0.209	-0.42	0.40
Snellen's chart - Visual Acuity	Visual	−0.17	0.209	−0.58	0.24
VFS	Visual	0.61	0.20	0.22	1.00
VSC	Visual	0.58	0.20	0.19	0.97
Autorefraction equipment- Refractive Power – Right	Visual	-2.18	0.25	-2.67	-1.69
Autorefraction equipment- Refractive Power – Left	Visual	-2.44	0.27	-2.97	-1.91
Blinking Rate	Psychophysiological	1.39	0.206	0.99	1.79
Depth Perception	Psychomotor	1.58	0.52	0.56	2.60
HR	Psychophysiological	-0.39	0.24	N-0.86	0.08
MASQ	Psychological	0.68	0.19	0.31	1.05
SBP	Psychophysiological	0.08	0.26	−0.43	0.59
HRV	Psychophysiological	0.37	0.26	-0.14	0.88
RR	Psychophysiological	0.30	0.26	−0.21	0.81
EDA	Psychophysiological	1.56	0.21	1.15	1.97
HAS	Psychological	1.10	0.24	0.63	1.57
PSS	Psychosocial	0.59	0.24	0.12	1.06
GAD-7	Psychosocial	0.41	0.24	−0.06	0.88
KSRS	Psychosocial	0.59	0.24	0.12	1.06
WEMWBS	Psychosocial	0.83	0.25	0.34	1.32
PSS	Psychosocial	0.43	0.24	−0.04	0.90
PSQI	Psychosocial	0.57	0.24	0.10	1.04
OSI	Psychosocial	0.73	0.25	0.24	1.22
IOP Right	Psychophysiological	1.06	0.29	0.49	1.63
IOP Left	Psychophysiological	0.70	0.27	0.17	1.23
SBP	Psychophysiological	0.77	0.27	0.24	1.30
DBP	Psychophysiological	0.51	0.26	0.00	1.02

Pulse Rate	Psychophysiological	0.67	0.27	0.14	1.20
Respiratory Rate	Psychophysiological	0.82	0.28	0.27	1.37
FBG	Psychophysiological	0.52	0.26	0.01	1.03
MWQ	Psychological	0.52	0.20	0.13	0.91
SMAAS	Psychological	0.48	0.20	0.09	0.87
PSS	Psychological	2.49	0.59	1.33	3.65
SRT	Cognitive	0.47	0.45	-0.41	1.35
Visual Memory	Cognitive	3.97	0.80	2.40	5.54
Hand-eye coordination	Cognitive	1.04	0.47	0.12	1.96
Executive Function	Cognitive	0.83	0.46	−0.07	1.73
TMT-B	Cognitive	0.52	0.28	−0.03	1.07
SLCT	Cognitive	0.35	0.27	−0.18	0.88
SCWT	Cognitive	0.75	0.19	0.38	1.12
Stroop Color	Cognitive	0.21	0.22	−0.22	0.64
SCWT	Cognitive	1.34	0.24	0.87	1.81
Stroop Word	Cognitive	0.38	0.23	−0.07	0.83
Stroop Interference	Cognitive	0.01	0.22	−0.42	0.44
Chronoscope - Reaction Timer	Cognitive	1.00	0.38	0.26	1.75
LCT	Cognitive	3.21	0.68	1.88	4.54
**Total Scores**					
MEAN VISUAL SCORE		0.29	0.14		
MEAN PSYCHOPHYSIOLOGICAL SCORE		0.74	0.26		
COGNITION SCORE		1.08	0.37		

b) Within-study design: Individual outcome effect sizes (Hedges' g)

**Outcome**	**Domain**	**Hedges' g**	**SE**	**95% CI Lower**	**95% CI Upper**
Asthenopia score	Visual	1.677	0.4	0.89	2.46
CVS-Q	Visual	0.968	0.266	0.44	1.48
CVS-Q	Visual	0.801	0.207	0.39	1.20
MWQ	Cognition	0.628	0.165	0.30	0.95
Stroop composite	Cognition	0.884	0.22	0.45	1.31
SLCT+DLST composite	Cognition	1.741	0.293	1.16	2.31
Corsi composite	Cognition	0.53	0.165	0.20	0.85
Shooting performance	Psychophysiological	4.051	0.691	2.69	5.40
Sinha's Comprehensive Anxiety Test	Psychophysiological	3.663	0.397	2.88	4.44
ISI	Psychophysiological	2.124	0.336	1.46	2.78
PSQI	Psychophysiological	1.415	0.262	0.90	1.92
Sinha's Comprehensive Anxiety Test	Psychophysiological	1.147	0.233	0.69	1.60
Critical Flicker Fusion Test	Psychophysiological	0.444	0.187	0.07	0.81
ECG – HRV	Psychophysiological	0.609	0.199	0.21	0.99
VEP -P100 Latency (L)	Psychophysiological	-0.167	0.202	-0.56	0.22
VEP-P100 Latency (R)	Psychophysiological	-0.183	0.19	-0.55	0.18

**Total Scores**		
MEAN VISUAL SCORE	1.149	0.291
MEAN PSYCHOPHYSIOLOGICAL SCORE	1.644	0.211
MEAN COGNITION SCORE	0.946	0.315

Supplementary Table S4 Notation and parameters used in statistical analyses

Parameter/Symbol	Common name	Description
M_1, M_2	Group means	M_1 – Intervention group, M_2 – Control group
SD_1, SD_2 group	Group standard deviations	SD_1 – Intervention group, SD_2 – Control
M_{pre}, M_{post} group	Pre-intervention	M_{pre} - Pre intervention group, M_{post} – post-intervention group
n_1, n_2	Group sample sizes	Number of participants in each group
N	Total sample size	Total participants across both groups
SD_{pooled}	Pooled standard deviation	Weighted average of group SDs, used as the denominator in SMD
d (Cohen's d)	Raw standardized mean difference	Difference between means in pooled SD units (biased in small samples)
J	Bias correction factor (Hedges)	Small-sample correction factor to reduce bias in d
g (Hedges' g)	Bias-corrected standardized mean difference (SMD)	Preferred SMD estimator in meta-analyses and small samples
SE	Standard error of the SMD	Measures the precision of the SMD estimate
95% CI	95% confidence intervals	Range likely containing the true population SMD
t	Standardization paired effect	Paired-samples t-statistic
d_z	Mean change	Standardized mean change based on paired t-statistic
Lower limit	Lower bound of CI	Smaller value of the CI range
Upper limit	Upper bound of CI	Larger value of the CI range
w_i	Study weight (Inverse-variance)	Precision-based weight for study in random-effects model
v_i	Within-study variance	Variance due to sampling error in the individual study

t^2	Between-study variance (heterogeneity)	Amount of true variation in effect sizes across studies
χ^2	Chi-squared test statistic	Measures deviation between observed and expected frequencies
Q	Cochran's Q Statistic	Test statistic for heterogeneity (follows chi distribution)
I^2	I-squared heterogeneity	Percentage of total variability due to between-study differences
k	Number of studies	Total studies included
$\hat{\mu}$ (pooled SMD/g)	Pooled effect size	Weighted average effect size across studies
SE_{HKSJ}	Hartung-Knapp-Sidik-Jonkman adjusted SE	Adjusted SE accounting for τ^2 estimation uncertainty (CI precision)
df	Degrees of freedom (chi-square)	Determine the critical value from the χ^2 distribution
t	t statistic or value	Estimated value by standard error
β	Effect size	Change in SMD of individual study

Supplementary Table S5: Grade Scale to assess the Certainty of Evidence

Outcome	Study-design	K = No of studies	Funnel/Egger's test (K≥10)	SMD (95% CI, I^2	Certainty of Evidence [GRADE]
Visual	RCT (Between-subject)	5	No	0.37 (-1.82, 2.57), 98%	Very Low
Cognitive	RCT (Between-subject)	3	No	0.78 (0.20, 1.35), 0%	Very Low
Psychophysiological	RCT (Between-subject)	6	No	1.08 (0.10, 2.06), 86%	Very Low
Visual	Non-randomized (Within-subject)	4	No	0.96 (-0.32, 2.25), 86%	Very Low
Cognitive	Non-randomized (Within-subject)	10	Borderline	0.85 (0.32, 1.37), 85%	Very Low
Psychophysiological	Non-randomized (Within-subject)	8	No	1.41 (0.40, 2.43), 93%	Very Low

No funnel plot or Egger's test was performed for any outcome due to the small number of studies. As recommended in the Cochrane Handbook (Sterne et al., 2011), the tests can be performed or interpreted when K≥10. All six outcomes showed very low certainty due to lack of methodological rigor, imprecision of pooled estimates, and substantial heterogeneity in RCTs (I^2 ranging from 0% to 98%) and in non-randomized studies (I^2 ranging from 85% to 93%). The final certainty of evidence was very low across all six domains (visual, cognitive, and psychophysiological domains, between-study and within-study) due to serious RoB, variation in participant age, intervention duration and frequency, and outcome

instruments.

Supplementary Table S6: Leave-one-out (LOO) analysis table for between- and within -subject studies.

Domain Design	Study removed	Full SMD	LOO SMD	LOO CI Lower	LOO CI Upper	Delta change and effect
Visual/Between-subject	Desai et al., 2020	0.47	1.04	0.2	1.87	0.56/ Yes
Visual/Between-subject	Telles et al., 2006	0.47	0.07	-1.18	1.32	-0.4/ No
Cognition/Within-subject	Sahana & Vijaya Kumar 2022	0.91	0.65	0.24	1.05	-0.26/Yes
Cognition/Within-subject	Parmar & Das 2025	0.99	0.69	0.41	0.97	-0.29/ No
Psychophysiological/ Within-subject	Kumar &Padmakar 2023	1.57	1.21	-0.19	2.62	-0.36/ No
Psychophysiological/ Within-subject	R.Tripathi 2024	1.57	1.21	-0.28	2.69	-0.37/ No
Cognition/Within-subject	Garg 2023	0.91	1.02	-0.51	2.54	0.11/ No
Psychophysiological/ Within-subject	Sushma &Dixit 2021	1.57	1.87	0.27	3.47	0.30/ Yes
Cognition/Between-subject	Sherlee & David 2020	0.99	1.17	0.53	1.8	0.18/ No
Cognition/Between-subject	Talwadkar et.al 2014	0.99	1.16	0.55	1.76	0.17/ No
Psychophysiological/ Within- subject	Bhat & Ramamurthy 2014	1.36	1.53	0.79	2.27	0.17/ No
Cognition/Between-subject	Pandya 2024	0.99	1.13	0.44	1.83	0.15/ No
Visual/Between-subject	Tripathi &Bhatnagar 2012	0.47	0.33	-1.24	1.9	-0.14/ No
Cognition/Within-subject	Swathi et al. 2021	0.91	1.05	-0.37	2.47	0.14/ No
Psychophysiological/ Within- subject	Mallick & Kulkarni 2010	1.57	1.75	-0.03	3.53	0.14/ No
Visual/Within-subject	Dhanesh Kumar K.U 2022	1.04	1.26	-3.17	5.69	0.22/ No
Visual/Between-subject	Tiwari et al., 2018	0.47	0.59	-0.94	2.12	0.11/ No
Cognition/Between-subject	Parmar & Das 2025	0.84	0.72	0.35	1.1	-0.11/ No
Psychophysiological/ Between -subject	Sankalp et al., 2022	0.84	0.95	0.63	1.28	0.11/ No
Cognition/Between-subject	Sukladas 2023	0.47	0.37	-1.11	1.86	-0.1/ No
Cognition/Between-subject	Ghosal & Kabi 2024	0.99	0.9	0.38	1.42	-0.08/ No
Visual/Within-subject	Soumya et al., 2017	1.04	0.86	-0.16	1.89	-0.18/ No
Visual/Within-subject	Wakhande et al., 2024	1.04	1.17	-4.33	6.67	0.13/ No
Psychophysiological/ Between -subject	Kusuma et al., 2021	0.84	0.91	0.48	1.33	0.07/ No
Psychophysiological/ Between -subject	Tripathi & Bhatnagar 2012	0.84	0.77	0.36	1.18	-0.07/ No
Psychophysiological/ Between- subject	Ghosal & Kabi 2024	0.84	0.78	0.37	1.19	-0.06/ No
Visual/Between-subject	Swathi et al., 2022	0.84	0.89	0.44	1.34	0.05/ No
Psychophysiological/ Within- subject	Rajpoot 2021	1.57	1.66	-0.17	3.49	0.09/ No
Psychophysiological/ Between- subject	Bhogal et al., 2025	0.84	0.87	0.43	1.32	0.04/ No
Psychophysiological/ Between- subject	Sherlee & David 2020	0.84	0.81	0.38	1.24	-0.03/ No

Psychophysiological/ Between- subject	Pandya 2024	0.84	0.81	0.37	1.24	-0.03/ No
Psychophysiological/ Between- subject	Swathi et al., 2022	0.47	0.45	-1.23	2.13	-0.02/ No
Cognition/Between-subject	Sukladas 2023	0.99	1.01	0.43	1.59	0.02/ No
Cognition/Within-subject	Raghavendra & Singh 2016	0.91	0.93	-0.7	2.56	0.03/No
Psychophysiological/ Between -subject	Ismail et al., 2022	0.84	0.86	0.42	1.3	0.02/ No
Psychophysiological/ Within -subject	Shathirapathiy et al., 2022	1.57	1.55	-0.29	3.4	-0.02/ No

Supplementary Figure S3: Leave-one-out (LOO) analysis Forest plot for between- and within -subject studies

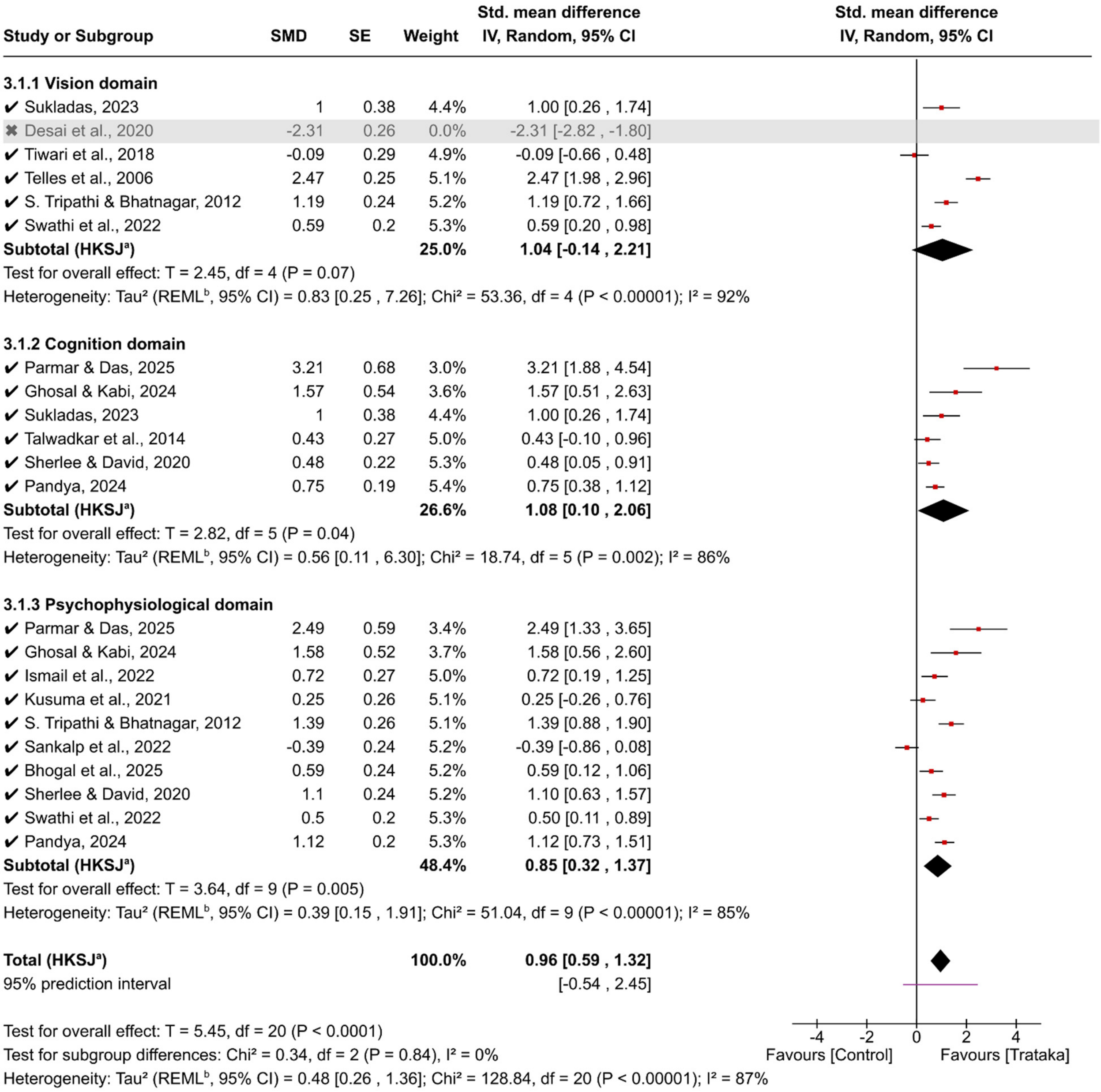

Study or Subgroup	SMD	SE	Weight	Std. mean difference IV, Random, 95% CI
**4.1.1 Vision domain**				
✓ Soumya et al., 2017	1.677	0.4	7.3%	1.68 [0.89 , 2.46]
✓ Wankhede et al., 2024	0.968	0.26	7.8%	0.97 [0.46 , 1.48]
✓ Dhanesh Kumar K.U. et al., 2022	0.801	0.2	8.0%	0.80 [0.41 , 1.19]
**Subtotal (HKSJ[a])**			**23.1%**	**1.04 [0.03 , 2.05]**
Test for overall effect: T = 4.43, df = 2 (P = 0.05)				
Heterogeneity: Tau^2 (REML[b], 95% CI) = 0.06 [0.00 , 8.42]; Chi^2 = 3.84, df = 2 (P = 0.15); I^2 = 45%				
**4.1.2 Cognition domain**				
✖ Sahana & Vijaya Kumar, 2022	1.741	0.293	0.0%	1.74 [1.17 , 2.32]
✓ Raghavendra & Singh, 2016	0.884	0.2	8.0%	0.88 [0.49 , 1.28]
✓ Garg, 2023	0.628	0.165	8.1%	0.63 [0.30 , 0.95]
✓ Swathi et al., 2021	0.53	0.165	8.1%	0.53 [0.21 , 0.85]
**Subtotal (HKSJ[a])**			**24.1%**	**0.66 [0.23 , 1.08]**
Test for overall effect: T = 6.66, df = 2 (P = 0.02)				
Heterogeneity: Tau^2 (REML[b], 95% CI) = 0.00 [0.00 , 1.28]; Chi^2 = 1.91, df = 2 (P = 0.38); I^2 = 0%				
**4.1.3 Psychophysiological domain**				
✓ Kumar & Padmakar, 2023	4.05	0.69	5.9%	4.05 [2.70 , 5.40]
✓ R. Tripathi, 2024	3.66	0.39	7.3%	3.66 [2.90 , 4.42]
✓ Shathirapathiy et al., 2022	1.76	0.29	7.7%	1.76 [1.19 , 2.33]
✓ Rajpoot, 2021	1.14	0.23	7.9%	1.14 [0.69 , 1.59]
✓ Bhat & Ramamurthy, 2014	0.6	0.19	8.0%	0.60 [0.23 , 0.97]
✓ Sushma & Dixit, 2021	-0.17	0.19	8.0%	-0.17 [-0.54 , 0.20]
✓ Mallick & Kulkarni, 2010	0.44	0.18	8.0%	0.44 [0.09 , 0.79]
**Subtotal (HKSJ[a])**			**52.8%**	**1.57 [0.09 , 3.05]**
Test for overall effect: T = 2.60, df = 6 (P = 0.04)				
Heterogeneity: Tau^2 (REML[b], 95% CI) = 2.35 [0.90 , 12.67]; Chi^2 = 120.28, df = 6 (P < 0.00001); I^2 = 98%				
**Total (HKSJ[a])**			**100.0%**	**1.23 [0.52 , 1.93]**
95% prediction interval				[-1.23 , 3.68]

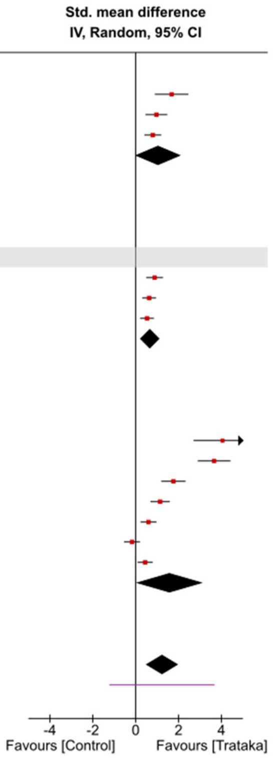

Test for overall effect: T = 3.76, df = 12 (P = 0.003)
Test for subgroup differences: Chi^2 = 4.22, df = 2 (P = 0.12), I^2 = 52.6%
Heterogeneity: Tau^2 (REML[b], 95% CI) = 1.16 [0.57 , 3.95]; Chi^2 = 129.30, df = 12 (P < 0.00001); I^2 = 96%

Study or Subgroup	SMD	SE	Weight	Std. mean difference IV, Random, 95% CI
**4.1.1 Vision domain**				
✓ Soumya et al., 2017	1.677	0.4	6.9%	1.68 [0.89 , 2.46]
✓ Wankhede et al., 2024	0.968	0.26	7.6%	0.97 [0.46 , 1.48]
✓ Dhanesh Kumar K.U. et al., 2022	0.801	0.2	7.9%	0.80 [0.41 , 1.19]
**Subtotal (HKSJ[a])**			**22.4%**	**1.04 [0.03 , 2.05]**
Test for overall effect: T = 4.43, df = 2 (P = 0.05)				
Heterogeneity: Tau^2 (REML[b], 95% CI) = 0.06 [0.00 , 8.42]; Chi^2 = 3.84, df = 2 (P = 0.15); I^2 = 45%				
**4.1.2 Cognition domain**				
✓ Sahana & Vijaya Kumar, 2022	1.741	0.293	7.5%	1.74 [1.17 , 2.32]
✓ Raghavendra & Singh, 2016	0.884	0.2	7.9%	0.88 [0.49 , 1.28]
✓ Garg, 2023	0.628	0.165	8.0%	0.63 [0.30 , 0.95]
✓ Swathi et al., 2021	0.53	0.165	8.0%	0.53 [0.21 , 0.85]
**Subtotal (HKSJ[a])**			**31.5%**	**0.90 [0.07 , 1.74]**
Test for overall effect: T = 3.44, df = 3 (P = 0.04)				
Heterogeneity: Tau^2 (REML[b], 95% CI) = 0.21 [0.03 , 4.15]; Chi^2 = 14.16, df = 3 (P = 0.003); I^2 = 85%				
**4.1.3 Psychophysiological domain**				
✖ Kumar & Padmakar, 2023	4.05	0.69	0.0%	4.05 [2.70 , 5.40]
✓ R. Tripathi, 2024	3.66	0.39	6.9%	3.66 [2.90 , 4.42]
✓ Shathirapathiy et al., 2022	1.76	0.29	7.5%	1.76 [1.19 , 2.33]
✓ Rajpoot, 2021	1.14	0.23	7.8%	1.14 [0.69 , 1.59]
✓ Bhat & Ramamurthy, 2014	0.6	0.19	8.0%	0.60 [0.23 , 0.97]
✓ Sushma & Dixit, 2021	-0.17	0.19	8.0%	-0.17 [-0.54 , 0.20]
✓ Mallick & Kulkarni, 2010	0.44	0.18	8.0%	0.44 [0.09 , 0.79]
**Subtotal (HKSJ[a])**			**46.1%**	**1.21 [-0.19 , 2.61]**
Test for overall effect: T = 2.22, df = 5 (P = 0.08)				
Heterogeneity: Tau^2 (REML[b], 95% CI) = 1.68 [0.61 , 10.93]; Chi^2 = 97.71, df = 5 (P < 0.00001); I^2 = 97%				
**Total (HKSJ[a])**			**100.0%**	**1.09 [0.53 , 1.64]**
95% prediction interval				[-0.86 , 3.04]

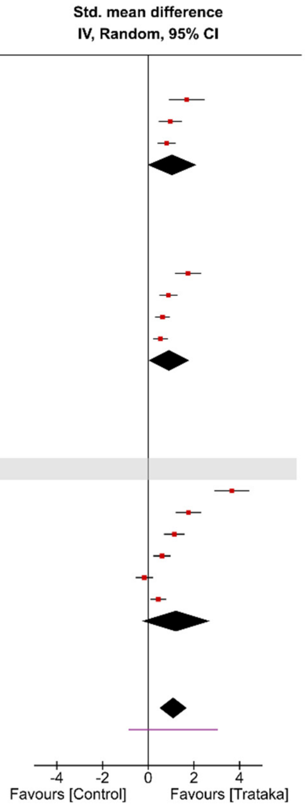

Test for overall effect: T = 4.27, df = 12 (P = 0.001)
Test for subgroup differences: Chi^2 = 0.31, df = 2 (P = 0.86), I^2 = 0%
Heterogeneity: Tau^2 (REML[b], 95% CI) = 0.74 [0.35 , 2.32]; Chi^2 = 117.56, df = 12 (P < 0.00001); I^2 = 94%

Study or Subgroup	SMD	SE	Weight	Std. mean difference IV, Random, 95% CI
**4.1.1 Vision domain**				
✔ Soumya et al., 2017	1.677	0.4	6.8%	1.68 [0.89 , 2.46]
✔ Wankhede et al., 2024	0.968	0.26	7.8%	0.97 [0.46 , 1.48]
✔ Dhanesh Kumar K.U. et al., 2022	0.801	0.2	8.1%	0.80 [0.41 , 1.19]
**Subtotal (HKSJ[a])**			**22.7%**	**1.04 [0.03 , 2.05]**
Test for overall effect: T = 4.43, df = 2 (P = 0.05)				
Heterogeneity: Tau² (REML[b], 95% CI) = 0.06 [0.00 , 8.42]; Chi² = 3.84, df = 2 (P = 0.15); I² = 45%				
**4.1.2 Cognition domain**				
✔ Sahana & Vijaya Kumar, 2022	1.741	0.293	7.6%	1.74 [1.17 , 2.32]
✔ Raghavendra & Singh, 2016	0.884	0.2	8.1%	0.88 [0.49 , 1.28]
✔ Garg, 2023	0.628	0.165	8.3%	0.63 [0.30 , 0.95]
✔ Swathi et al., 2021	0.53	0.165	8.3%	0.53 [0.21 , 0.85]
**Subtotal (HKSJ[a])**			**32.4%**	**0.90 [0.07 , 1.74]**
Test for overall effect: T = 3.44, df = 3 (P = 0.04)				
Heterogeneity: Tau² (REML[b], 95% CI) = 0.21 [0.03 , 4.15]; Chi² = 14.16, df = 3 (P = 0.003); I² = 85%				
**4.1.3 Psychophysiological domain**				
✔ Kumar & Padmakar, 2023	4.05	0.69	4.7%	4.05 [2.70 , 5.40]
✖ R. Tripathi, 2024	3.66	0.39	0.0%	3.66 [2.90 , 4.42]
✔ Shathirapathiy et al., 2022	1.76	0.29	7.6%	1.76 [1.19 , 2.33]
✔ Rajpoot, 2021	1.14	0.23	8.0%	1.14 [0.69 , 1.59]
✔ Bhat & Ramamurthy, 2014	0.6	0.19	8.2%	0.60 [0.23 , 0.97]
✔ Sushma & Dixit, 2021	-0.17	0.19	8.2%	-0.17 [-0.54 , 0.20]
✔ Mallick & Kulkarni, 2010	0.44	0.18	8.2%	0.44 [0.09 , 0.79]
**Subtotal (HKSJ[a])**			**44.9%**	**1.20 [-0.28 , 2.69]**
Test for overall effect: T = 2.09, df = 5 (P = 0.09)				
Heterogeneity: Tau² (REML[b], 95% CI) = 1.69 [0.57 , 13.13]; Chi² = 63.52, df = 5 (P < 0.00001); I² = 97%				
**Total (HKSJ[a])**			**100.0%**	**1.04 [0.50 , 1.58]**
95% prediction interval				[-0.68 , 2.76]

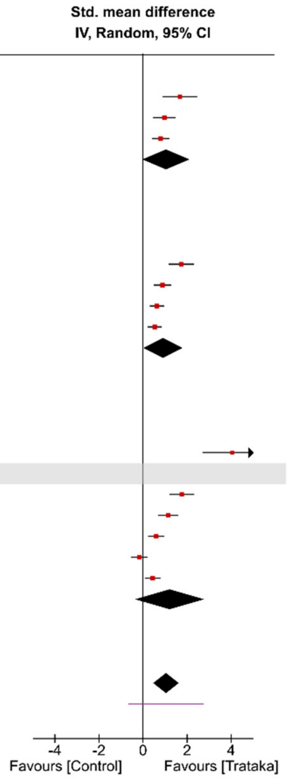

Test for overall effect: T = 4.19, df = 12 (P = 0.001)
Test for subgroup differences: Chi² = 0.28, df = 2 (P = 0.87), I² = 0%
Heterogeneity: Tau² (REML[b], 95% CI) = 0.56 [0.29 , 2.60]; Chi² = 85.27, df = 12 (P < 0.00001); I² = 92%

Supplementary Figure S3: Leave-one-out sensitivity analysis showed that the pooled effects remained positive across both study designs. In within-subject analyses, the largest changes were observed after removal of R. Tripathi (Δ = −0.37), Kumar and Padmakar (Δ = −0.36), and Sushma and Dixit (Δ = +0.30) [4,5,38,44,57,58,112,115,117,123,125], while in between-subject analyses, removal of Desai et al. produced the largest increase (Δ = +0.56) [3, 67,81,107,109,110,113,114,118,120,121,122,124,126]. Despite these variations, no individual study reversed the direction of the pooled effect

Supplementary Figure S4 : Only RCT - sensitivity analysis

To examine the robustness of the findings to study design, an RCT-only sensitivity analysis was performed (Supplementary Fig. S4). Among the included studies, 12 were randomized controlled trials, and 23 were non-randomized or quasi-experimental. Of these 12, one did not report sufficient statistical information to compute SMD and therefore was not included in the quantitative synthesis as mentioned in the methods earlier. So, a sensitivity analysis for the11 RCTs alone was performed (Supplementary Fig. S4). In the RCT-only analysis, the psychophysiological domain favoured Trataka with statistical significance (SMD =0.68, 95% CI 0.04-1.32, I² =87%), whereas the cognitive effect was positive but not statistically significant (SMD =1.02, 95% CI 0.25-2.29, I² = 90%). The vision domain showed no significant effect and substantial heterogeneity (SMD =0.86, 95%, I² = 99%). Overall, the pooled RCT estimate was positive with statistical insignificance (SMD =0.62, 95% CI 0.03-1.26, I² = 95%), with a wide predicted interval (-1.78 to 3.02). [3, 67,81,112,114,119,121,124,125,127,129]

Study or Subgroup	SMD	SE	Weight	Std. mean difference IV, Random, 95% CI
**5.1.1 Vision domain**				
Desai et al., 2020	-2.31	0.26	6.8%	-2.31 [-2.82 , -1.80]
Swathi et al., 2022	0.59	0.2	7.0%	0.59 [0.20 , 0.98]
**Subtotal (HKSJ[a])**			**13.8%**	**-0.86 [-19.28 , 17.57]**
Test for overall effect: T = 0.59, df = 1 (P = 0.66)				
Heterogeneity: Tau^2 (REML[b], 95% CI) = 4.15 [0.78 , >100]; Chi^2 = 78.16, df = 1 (P < 0.00001); I^2 = 99%				
**5.1.2 Cognition domain**				
Parmar & Das, 2025	3.21	0.68	5.2%	3.21 [1.88 , 4.54]
Sukladas, 2023	1	0.38	6.4%	1.00 [0.26 , 1.74]
Talwadkar et al., 2014	0.43	0.27	6.8%	0.43 [-0.10 , 0.96]
Sherlee & David, 2020	0.48	0.22	6.9%	0.48 [0.05 , 0.91]
Pandya, 2024	0.75	0.19	7.0%	0.75 [0.38 , 1.12]
**Subtotal (HKSJ[a])**			**32.3%**	**1.02 [-0.25 , 2.29]**
Test for overall effect: T = 2.23, df = 4 (P = 0.09)				
Heterogeneity: Tau^2 (REML[b], 95% CI) = 0.70 [0.13 , 10.76]; Chi^2 = 16.31, df = 4 (P = 0.003); I^2 = 90%				
**5.1.3 Psychophysiological domain**				
Parmar & Das, 2025	2.49	0.59	5.6%	2.49 [1.33 , 3.65]
Ismail et al., 2022	0.72	0.27	6.8%	0.72 [0.19 , 1.25]
Kusuma et al., 2021	0.25	0.26	6.8%	0.25 [-0.26 , 0.76]
Sankalp et al., 2022	-0.39	0.24	6.9%	-0.39 [-0.86 , 0.08]
Sherlee & David, 2020	1.1	0.24	6.9%	1.10 [0.63 , 1.57]
Bhogal et al., 2025	0.59	0.24	6.9%	0.59 [0.12 , 1.06]
Pandya, 2024	1.12	0.2	7.0%	1.12 [0.73 , 1.51]
Swathi et al., 2022	0.25	0.2	7.0%	0.25 [-0.14 , 0.64]
**Subtotal (HKSJ[a])**			**53.8%**	**0.68 [0.04 , 1.32]**
Test for overall effect: T = 2.51, df = 7 (P = 0.04)				
Heterogeneity: Tau^2 (REML[b], 95% CI) = 0.42 [0.15 , 2.80]; Chi^2 = 43.40, df = 7 (P < 0.00001); I^2 = 87%				
**Total (HKSJ[a])**			**100.0%**	**0.62 [-0.03 , 1.26]**
95% prediction interval				[-1.78 , 3.02]

Test for overall effect: T = 2.06, df = 14 (P = 0.06)
Test for subgroup differences: Chi^2 = 1.63, df = 2 (P = 0.44), I^2 = 0%
Heterogeneity: Tau^2 (REML[b], 95% CI) = 1.16 [0.59 , 3.46]; Chi^2 = 179.95, df = 14 (P < 0.00001); I^2 = 95%

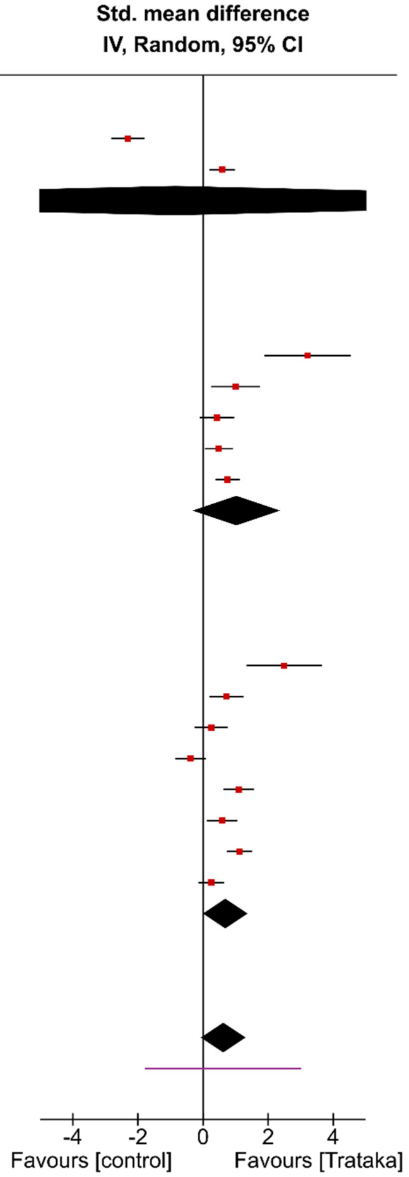

Supplementary Figure S5: Bubble plot by including one study in between-subject study analysis

A sensitivity analysis by including this study (Parmar & Das, 2025) in the between-subject regression (K=5) was conducted. The slope reversed in direction(β =-0.009 vs = β 0.018 when excluded) and became further from significance (P =0.7891 vs P =0.2201), while the variance explained by duration fell from R =0.608 to R =0.028. This instability demonstrates that a single excluded study exerts disproportionate influence on the model, a high-leverage outlier rather than a data point that extends an underlying trend, suggesting no robust duration-response relationship can be inferred. Both regression outputs have no significant difference, but the effect size of SMD= 3.21 falls outside the range of the 95% prediction interval of the regression model fitted to the remaining four studies.

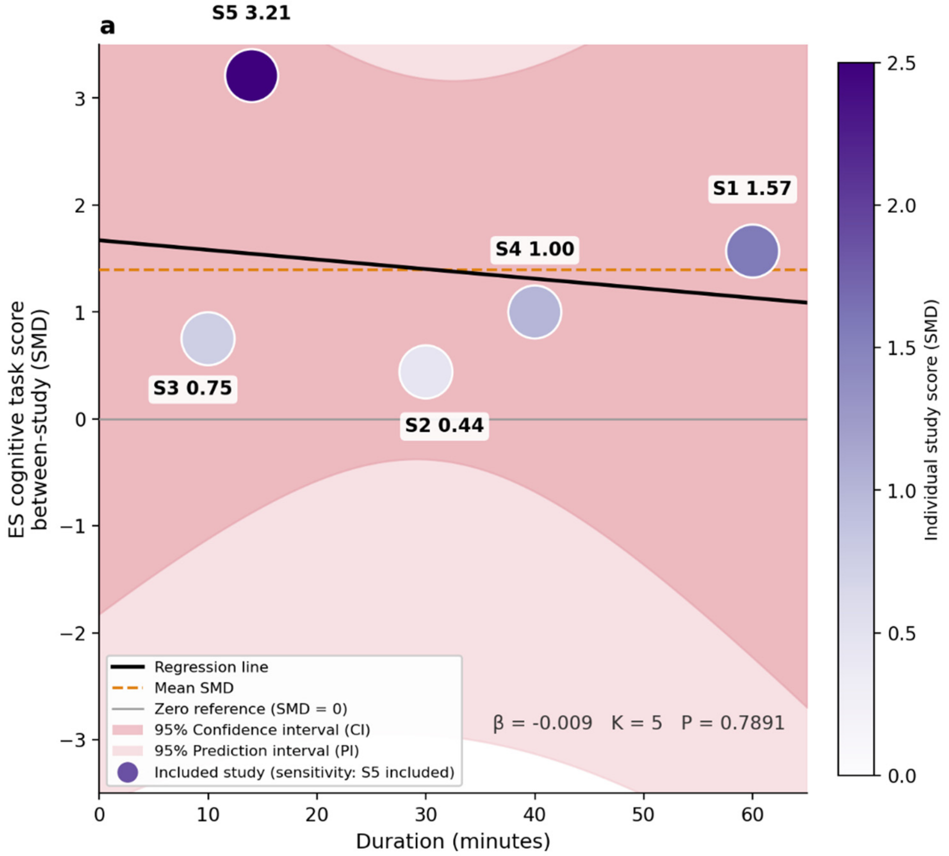

Supplementary Table S7: Operational definitions for the usage of different key terms in the studies.

Term	Operational definition for this systematic review
Trataka	A yogic visual concentration practice involving sustained or focused gazing at a specified object or target, as described in the individual included studies.
Intervention	The Trataka/YVC-based practice or program evaluated in an included study, including its reported duration, frequency, and mode of delivery where available.
Technique	A specific procedural method or component used within a Trataka/YVC practice, as described by the authors of an included study.
Practice	The performance of Trataka, YVC, or related visual concentration exercises by participants within an included study.
YVC	Yogic Visual Concentration (YVC), referring to visual concentration practices derived from or incorporating Trataka and described as such in the included studies.

Supplementary Table S8: PRISMA checklist

Section and Topic	Item #	Checklist item	Location where item is reported
**TITLE**			
Title	1	Identify the report as a systematic review.	1
**ABSTRACT**			
Abstract	2	See the PRISMA 2020 for Abstracts checklist.	1
**INTRODUCTION**			
Rationale	3	Describe the rationale for the review in the context of existing knowledge.	3
Objectives	4	Provide an explicit statement of the objective(s) or question(s) the review addresses.	3
**METHODS**			
Eligibility criteria	5	Specify the inclusion and exclusion criteria for the review and how studies were grouped for the syntheses.	7
Information sources	6	Specify all databases, registers, websites, organisations, reference lists and other sources searched or consulted to identify studies. Specify the date when each source was last searched or consulted.	4-5
Search strategy	7	Present the full search strategies for all databases, registers and websites, including any filters and limits used.	4-5
Selection process	8	Specify the methods used to decide whether a study met the inclusion criteria of the review, including how many reviewers screened each record and each report retrieved, whether they worked independently, and if applicable, details of automation tools used in the process.	4-6
Data collection process	9	Specify the methods used to collect data from reports, including how many reviewers collected data from each report, whether they worked independently, any processes for obtaining or confirming data from study investigators, and if applicable, details of automation tools used in the process.	7
Data items	10a	List and define all outcomes for which data were sought. Specify whether all results that were compatible with each outcome domain in each study were sought (e.g. for all measures, time points, analyses), and if not, the methods used to decide which results to collect.	8 -11
	10b	List and define all other variables for which data were sought (e.g. participant and intervention characteristics, funding sources). Describe any assumptions made about any missing or unclear information.	10
Study risk of bias assessment	11	Specify the methods used to assess risk of bias in the included studies, including details of the tool(s) used, how many reviewers assessed each study and whether they worked independently, and if applicable, details of automation tools used in the process.	9
Effect measures	12	Specify for each outcome the effect measure(s) (e.g. risk ratio, mean difference) used in the synthesis or presentation of results.	9
Synthesis	13a	Describe the processes used to decide which studies were eligible for	9-10

Section and Topic	Item #	Checklist item	Location where item is reported
methods		each synthesis (e.g. tabulating the study intervention characteristics and comparing against the planned groups for each synthesis (item #5)).	
	13b	Describe any methods required to prepare the data for presentation or synthesis, such as handling of missing summary statistics, or data conversions.	10-11
	13c	Describe any methods used to tabulate or visually display results of individual studies and syntheses.	11-12
	13d	Describe any methods used to synthesize results and provide a rationale for the choice(s). If meta-analysis was performed, describe the model(s), method(s) to identify the presence and extent of statistical heterogeneity, and software package(s) used.	11-12
	13e	Describe any methods used to explore possible causes of heterogeneity among study results (e.g. subgroup analysis, meta-regression).	12
	13f	Describe any sensitivity analyses conducted to assess robustness of the synthesized results.	na
Reporting bias assessment	14	Describe any methods used to assess risk of bias due to missing results in a synthesis (arising from reporting biases).	RoB and ROBINS I
Certainty assessment	15	Describe any methods used to assess certainty (or confidence) in the body of evidence for an outcome.	S5 table
**RESULTS**			
Study selection	16a	Describe the results of the search and selection process, from the number of records identified in the search to the number of studies included in the review, ideally using a flow diagram.	6-7
	16b	Cite studies that might appear to meet the inclusion criteria, but which were excluded, and explain why they were excluded.	7
Study characteristics	17	Cite each included study and present its characteristics.	S1 table
Risk of bias in studies	18	Present assessments of risk of bias for each included study.	S1figure
Results of individual studies	19	For all outcomes, present, for each study: (a) summary statistics for each group (where appropriate) and (b) an effect estimate and its precision (e.g. confidence/credible interval), ideally using structured tables or plots.	S3 and s4 table
Results of syntheses	20a	For each synthesis, briefly summarise the characteristics and risk of bias among contributing studies.	S1
	20b	Present results of all statistical syntheses conducted. If meta-analysis was done, present for each the summary estimate and its precision (e.g. confidence/credible interval) and measures of statistical heterogeneity. If comparing groups, describe the direction of the effect.	S3 and S4 11-12
	20c	Present results of all investigations of possible causes of heterogeneity among study results.	11, 18
	20d	Present results of all sensitivity analyses conducted to assess the robustness of the synthesized results.	S4

Section and Topic	Item #	Checklist item	Location where item is reported
Reporting biases	21	Present assessments of risk of bias due to missing results (arising from reporting biases) for each synthesis assessed.	14-15
Certainty of evidence	22	Present assessments of certainty (or confidence) in the body of evidence for each outcome assessed.	S5
**DISCUSSION**			
Discussion	23a	Provide a general interpretation of the results in the context of other evidence.	18-19
	23b	Discuss any limitations of the evidence included in the review.	1
	23c	Discuss any limitations of the review processes used.	20-22
	23d	Discuss implications of the results for practice, policy, and future research.	20-22
**OTHER INFORMATION**			
Registration and protocol	24a	Provide registration information for the review, including register name and registration number, or state that the review was not registered.	na
	24b	Indicate where the review protocol can be accessed, or state that a protocol was not prepared.	NA
	24c	Describe and explain any amendments to information provided at registration or in the protocol.	na
Support	25	Describe sources of financial or non-financial support for the review, and the role of the funders or sponsors in the review.	22
Competing interests	26	Declare any competing interests of review authors.	22
Availability of data, code and other materials	27	Report which of the following are publicly available and where they can be found: template data collection forms; data extracted from included studies; data used for all analyses; analytic code; any other materials used in the review.	6-11
